# The association between recent stressful life events and brain structure: a UK Biobank longitudinal MRI study

**DOI:** 10.1192/j.eurpsy.2025.2

**Published:** 2025-01-21

**Authors:** Cheryl R.Z. See, Annabel X. Tan, Lucia R. Valmaggia, Matthew J. Kempton

**Affiliations:** 1 Department of Psychosis Studies, Institute of Psychiatry, Psychology & Neuroscience, King’s College London, London, UK; 2 Department of Epidemiology and Population Health, Stanford University, Stanford, CA, USA; 3 Centre for Youth Mental Health, University of Melbourne, Parkville, VIC, Australia; 4 Department of Psychology, Institute of Psychiatry, Psychology & Neuroscience, King’s College London, London, UK; 5 Department of Psychiatry, KU Leuven, Leuven, Belgium

**Keywords:** gray matter, longitudinal, recent stress, structural neuroimaging

## Abstract

**Background:**

Recent stressful life events (SLEs) are an established risk factor for a range of psychiatric disorders. Animal studies have shown evidence of gray matter (GM) reductions associated with stress, and previous work has found similar associations in humans. However longitudinal studies investigating the association between stress and changes in brain structure are limited.

**Methods:**

The current study uses longitudinal data from the UK Biobank and comprises 4,543 participants with structural neuroimaging and recent SLE data (mean age = 61.5 years). We analyzed the association between recent SLEs and changes in brain structure, determined using the longitudinal FreeSurfer pipeline, focusing on total GM volume and five a priori brain regions: the hippocampus, amygdala, anterior cingulate cortex, orbitofrontal cortex, and insula. We also examined if depression and childhood adversity moderated the relationship between SLEs and brain structure.

**Results:**

Individuals who had experienced recent SLEs exhibited a slower rate of hippocampal decrease over time compared to individuals who did not report any SLEs. Individuals with depression exhibited smaller GM volumes when exposed to recent SLEs. There was no effect of childhood adversity on the relationship between SLEs and brain structure.

**Conclusions:**

Our findings suggest recent SLEs are not directly associated with an accelerated decline in brain volumes in a population sample of older adults, but instead may alter brain structure via affective disorder psychopathology. Further work is needed to investigate the effects of stress in younger populations who may be more vulnerable to stress-induced changes, and may yet pinpoint brain regions linked to stress-related disorders.

## Introduction

Stressful life events (SLEs) are a recognized risk factor for a range of disorders including depression, psychosis, and infectious illnesses [1]. The brain is central to responding to external stressors and regulating the biological stress response [2]. Findings from animal studies have suggested prolonged exposure to stress can cause reductions in gray matter (GM) volumes within the brain in areas such as the hippocampus, cingulate cortex, and prefrontal regions [3–5]. This has been attributed to the hypothalamic–pituitary–adrenal (HPA) axis, the main biological system that secretes glucocorticoids in response to stress, which is thought to be neurotoxic at sustained levels [6, 7]. It is posited this could in part be due to excitotoxicity, where the over-stimulation of cells via glutamate receptors are further exacerbated by elevated glucocorticoids, resulting in neuronal damage [8, 9].

Cross-sectional structural neuroimaging studies have reported associations between recent SLEs in adulthood and smaller gray matter (GM) volumes within regions including the anterior cingulate cortex (ACC), insula, prefrontal cortex, hippocampus, and amygdala in healthy adults [10–13]. To clarify the direction of effect, studies using longitudinal data are required, of which we are aware of only two in non-clinical samples. Papagni et al. [14] (*N* = 26) found reductions in the hippocampus, parahippocampus, and anterior cingulate cortex (ACC) associated with SLEs that occurred over a three-month period. Ringwald et al. [15] (*N* = 212) found a negative association between SLEs and GM volume changes over a 2-year follow-up period within the medial prefrontal cortex. These findings indicate that recent SLEs may have a detectable effect on macroscopic brain structure.

The current study investigated the effects of recent SLEs on brain structure using a large, longitudinal, population dataset from the UK Biobank (https://www.ukbiobank.ac.uk). Previous work examining the neural correlates of lifetime adulthood stress and early life adversity using cross-sectional UK Biobank data has been carried out by McManus et al. [16], where the authors did not find a significant association with GM volumes in their hypothesized regions-of-interest (ROIs): the hippocampus, amygdala, and thalamus. Here, we sought to investigate whether recent SLEs are distinctly associated with brain structural changes in this population sample. We have focused on total GM volume and five subregions of the brain: the hippocampus, amygdala, ACC, orbitofrontal cortex (OFC), and insula. These regions have previously been implicated in the regulation of the stress response [7], with evidence of structural neuroanatomical changes within these regions associated with stress in non-clinical adult samples [10–15, 17]. These brain regions have also been observed to exhibit aberrant functional connectivity associated with stress in healthy adults [18, 19], and in animal studies [20]. One study further observed persistent anomalous resting-state connectivity in rodents a week after being exposed to a stressor within the prefrontal cortex and amygdala [21].

We have analyzed a subset of UK Biobank participants who had available structural neuroimaging and recent SLE data measured at two timepoints. We categorized participants into two groups based on whether they had any or no recent SLE exposure and examined for group differences in brain structure. We hypothesized that individuals exposed to recent SLEs would have smaller brain volumes and lower cortical thickness, and that changes in their brain structure over the follow-up period would be more adversely affected when compared to individuals without recent SLE exposure. Among individuals with recent SLE exposure, we hypothesized that brain structural measures would be negatively associated with the number of events.

## Methods

### UK Biobank data

The UK Biobank is a population-based cohort of over 500,000 participants from across the United Kingdom (https://www.ukbiobank.ac.uk), recruited between the ages of 40 and 69 [22]. Recruitment began in 2006, and baseline data were collected covering an extensive range of variables relating to health and wellbeing, sociodemographic measures, and lifestyle. There have since been three follow-up assessments, where imaging data were collected in the latter two [23]. Between 2014 and 2020, participants completed their first MRI scan, while data collection for the second MRI scan occurred between 2019 and 2022. At each follow-up, participants who completed the MRI scan also completed the main assessment suite on the same day, which included recent SLE data and depressive symptom data. The current study used data from participants who had complete structural MRI data and recent SLE data at both imaging visits (*N* = 4,543). In between assessments, participants were also invited to complete one-off online questionnaires such as the 2016 Mental Health Questionnaire (see Supplementary Figure S1 for a timeline illustrating data collection). The current study obtained only childhood adversity data from the Mental Health Questionnaire. Supplementary Figure S2 presents a flow chart depicting sample sizes of the analyses and a list of the variables used is reported in Supplementary Table S1.

The UK Biobank obtained ethical approval from the Research Ethics Committee (Ref:11/NW/0382), and participants provided written, informed consent. Data in the current study (application ID: 87152) were retrieved from the UK Biobank in July 2023.

### Recent stressful life events

At each imaging assessment, participants were asked if they had experienced any SLEs within the last 2 years (Data-Field 6145). Participants selected events from a pre-specified list of six events which included: a serious illness, injury or assault to self or to a close relative, death of a close relative or spouse/partner, marital separation/divorce, or financial difficulties. We calculated an SLE score based on the number of events (0–6). Participants were assigned group membership at each timepoint to either SLE−, for scores of zero, or SLE+, for scores greater than zero. In our analyses, we compared brain structural measures between the two groups, and we also examined the association between the SLE score and brain structure within the SLE+ group.

### Neuroimaging measures

The UK Biobank’s MRI acquisition protocol and quality control have been previously described [24]. Participants were scanned at four centers (Cheadle, Reading, Newcastle, and Bristol) using the same scanner model (3 T Siemens Skyra). At the time of data retrieval in the current study, there were no repeat imaging scans completed at Bristol, and therefore only data from three centers have been included.

T1-weighted scans from both timepoints were processed using the longitudinal stream in the software FreeSurfer (v7.3.2) (https://surfer.nmr.mgh.harvard.edu) [25], which has demonstrated reliable structural measurements for longitudinal neuroimaging analysis [26]. Segmented regions were derived based on the Desikan–Killiany Atlas [27]. In the current study, we focused on global total GM volume and five brain regions that have been previously associated with recent stress in healthy adults: (1) hippocampus [14, 28, 29]; (2) amygdala [12, 29]; (3) OFC [10, 13]; (4) ACC [11, 13, 14]; and (5) insula [13, 17]. Results from the FreeSurfer processing were assessed following the ENIGMA Quality Control (QC) Protocol (https://enigma.ini.usc.edu/protocols/imaging-protocols/), where seven participants were excluded due to poor data quality. Further details are reported in the supplement.

For the subcortical regions, the hippocampus and amygdala, we analyzed bilateral GM volumes summing left and right volume measures as obtained from FreeSurfer. For the cortical regions, the OFC, ACC, and insula, we analyzed the mean cortical thickness, which was calculated by averaging the FreeSurfer thickness estimates across hemispheres for each region.

### Other non-imaging variables

#### Time

The time between assessments was considered as the time from the first imaging assessment and calculated using the assessment date (Data-Field 53) for each participant. Time at the first imaging assessment was therefore zero across all participants. The time to the second imaging assessment was calculated in days by subtracting assessment dates and dividing by 365 to convert it to years.

#### Depressive symptoms

Recent depressive symptoms were measured using the total score of the Patient Health Questionnaire (PHQ)-2 (Data-Fields 2050–2080), the depression subscale of the PHQ-4 [30, 31]. We selected the PHQ-2 as these data were collected on the same day as the MRI scans and were the most complete measure of psychopathology. As a large number of participants scored zero, indicating no recent depressive symptoms, we grouped participants based on the established PHQ-2 cut-off score, where scores of ≥ 3 indicated probable depression (PHQ+) and < 3 indicated no probable depression (PHQ−) [30].

#### Childhood adversity

Participants completed the Childhood Trauma Screener (CTS-5) [32] as part of the online 2016 Mental Health Questionnaire (Data-Fields 20489–20491), and a total CTS-5 score was calculated. Not all participants completed the online assessment, which was issued between follow-up assessments, and 1,219 participants were missing data. As just over half of the participants reported experiencing no childhood adversity, we created a childhood adversity (CA) grouping where participants were assigned membership based on whether they experienced any (CA+) or no childhood adversity (CA−).

#### Sociodemographic variables

Other variables used in the current study as potential confounders included: employment status (Data-Field 6142), the presence of a long-standing illness, disability, or infirmity (Data-Field 2188), alcohol intake frequency (Data-Field 1558), smoking status (Data-Field 20116), and the Townsend deprivation index (Data-Field 22189). The Townsend deprivation index measures socioeconomic deprivation, with higher scores indicating higher deprivation [33]. Further details regarding the treatment of the variables are reported in the supplement.

### Statistical analysis

All analyses were conducted in R (v4.3.1), with the statistical significance level set at *p* < 0.05 (two-tailed).

Sample characteristics of the SLE− and SLE+ groups were compared using independent sample *t*-tests or chi-square tests as appropriate, using data from the first imaging assessment or at recruitment.

Our primary analysis was to compare brain structural measures between the groups SLE+ and SLE−. We employed linear mixed models (LMM), using the R package *lme4* [34], and participants were modeled with random intercepts to account for the repeated measures. LMMs do not require data to be measured at consistent time intervals making it suitable for analyzing longitudinal data [35]. We fitted separate LMMs with each brain structural measure as the outcome variable, and SLE group, time, and the interaction term SLE group × time as the main fixed effects. The interaction term allowed us to examine whether there were group differences in brain structural changes over the study period. Time was measured in years from the date of the first imaging assessment.

In an additional analysis we fitted LMMs using SLE score with data from only the SLE+ group, to examine whether the number of SLEs were associated with changes in brain structure. Separate LMMs were modeled for each brain structural measure as the outcome variable, and with SLE score, time, and SLE score × time as the main fixed effects.

In exploratory analysis, we investigated whether recent SLEs influenced the relationship between depression and brain structure, given the strong evidence linking recent stress and the onset of depressive disorders [36, 37]. We fitted LMMs with depression group (PHQ+ or PHQ−), SLE group, and the interaction term SLE group × depression group as the main fixed effects, controlling for time. We also considered the effects of childhood adversity as it has been associated with smaller brain volumes [38, 39], and is linked to an increased sensitivity to stress in later life, potentially amplifying the effects of stress in adulthood [40, 41]. To examine for the effects of childhood adversity, we fitted LMMs to include CA group (CA+ or CA−), SLE group, and the interaction term CA group × SLE group as the main fixed effects, controlling for time.

In all models, where the interaction term was not significant, we re-fitted the models excluding the interaction term to report the fixed effects of the variables of interest [42]. All models were adjusted for age, age^2^ (where age was taken at the first imaging assessment), sex, total intracranial volume (ICV), and scan center, included as fixed effects. Age and total ICV were standardized to avoid varying scales across covariates affecting model convergence [34]. Neuroimaging and SLE data were used across both timepoints in all models.

To adjust for multiple comparisons, we used a 5% false discovery rate (FDR) correction inclusive of the main and exploratory analyses (51 *p*-values). The *p-*values reported in the results section are uncorrected, with a superscript indicating whether significant *p*-values had passed correction.

We conducted several sensitivity analyses to test for changes to the significance of our results in our main SLE group analysis. Firstly, we excluded data from participants who had experienced a stroke in their lifetime (*n* = 52), and who had outlier total ICV (*n* = 36), defined in the ENIGMA QC protocol as 2.698 standard deviations above or below the sample mean ICV. Next, we adjusted the models for potential confounding sociodemographic variables, which were found to be different between SLE groups (see Table 1): employment status, the presence of a long-standing illness, disability or infirmity, alcohol intake frequency, smoking status, and the Townsend deprivation index. Finally, we re-fit the models to include a broader range of neuroimaging confounders as identified by Alfaro-Almagro et al. [43], which included non-linear terms for time, age and sex interactions, and head motion measures. Further details are provided in the supplement.

**Table 1.** Sample characteristics of participants at the first imaging assessment (*n* = 4,543)

		SLE–	SLE+	SLE– versus SLE+	
Variable		(*n* = 2,794)	(*n* = 1,749)	*t*-Value/χ^2^	*p*-Value
Age, mean (SD)		62.20 (7.54)	60.39 (7.19)	8.01	1.45 × 10^−15^**
Sex, *n* (%)	Female	1,446 (51.8)	983 (56.2)	8.56	0.003**
Ethnicity, *n* (%)	White	2,716 (97.2)	1,697 (97.0)	3.76	0.584
	Mixed	10 (0.4)	6 (0.3)		
	Asian/Asian British	23 (0.8)	22 (1.3)		
	Black/Black British	17 (0.6)	13 (0.7)		
	Chinese	10 (0.4)	3 (0.2)		
	Other ethnic group	13 (0.5)	7 (0.4)		
Current employment status, *n* (%)	In employment	1269 (45.4)	956 (54.7)	63.66	4.92 × 10^−13^**
	Retired	1440 (51.5)	704 (40.3)		
	Unable to work	59 (2.1)	70 (4.0)		
	Unemployed	10 (0.4)	10 (0.6)		
	Other	14 (0.5)	5 (0.3)		
Alcohol intake frequency, *n* (%)	Daily	435 (15.6)	238 (13.6)	29.90	1.45 × 10^−6^**
	1–4 times/week	1701 (60.9)	979 (56.0)		
	<3 times/month	502 (18.0)	429 (24.5)		
	None	156 (5.6)	102 (5.8)		
Smoking status, *n* (%)	Current	75 (2.7)	70 (4.0)	8.20	0.017*
	Previous	921 (33.0)	533 (30.5)		
	Never	1789 (64.0)	1142 (65.3)		
Townsend Deprivation Index^a^, mean (SD)		−2.05 (2.59)	−1.77 (2.77)	−3.35	0.001**
Experienced any childhood adversity (CA+), *n* (%)		1195 (42.8)	749 (42.8)	0.32	0.570
Probable depression (PHQ+), mean (SD)		488 (17.5)	465 (26.6)	54.83	1.32 × 10^−13^**
Any long-standing illness, disability, or infirmity, *n* (%)		484 (17.32)	393 (22.47)	19.31	1.11 × 10^−5^**

Abbreviations: SLE, stressful life event; SLE−, no recent SLE; SLE+, one or more recent SLE; CA+, any childhood adversity; PHQ+, probable depression based on the Patient Health Questionnaire-4 depression subscale.

a Townsend deprivation index measures the socioeconomic deprivation of a participant’s census area (based on postcode) at point of recruitment. Higher scores indicate higher socioeconomic deprivation.

* *p* < 0.05.

** *p* < 0.01.

### Whole-brain exploratory analysis

We conducted a final exploratory analysis looking at group differences in structural measures across all FreeSurfer regions in the brain between SLE+ and SLE−. This analysis was to provide further insight into potential stress-affected brain regions separate from our analytical plan detailed above. Using LMMs with SLE group as the main fixed effect, and controlling for time, we examined left and right cortical thickness and surface area measures for a total of 68 regions, and left and right subcortical volumes for a total of 18 regions. Results were corrected for multiple comparisons using a 5% FDR correction.

## Results

### Sample characteristics

The current study used 4,543 participants from the UK Biobank who had available neuroimaging and recent SLE data at both imaging assessments. Sample characteristics and group differences between SLE+ and SLE- at the first imaging assessment are reported in Table 1. The SLE+ group were younger, consisted of more females, were more likely to be in employment, consumed less alcohol, more likely to be current smokers, and lived in more socioeconomically deprived areas. More SLE+ individuals reported having a long-standing illness, disability or infirmity and depression, and had a mean SLE score of 1.26 (SD = 0.53) at the first imaging assessment. The frequencies of SLE types are reported in Supplementary Table S2. All participants completed two imaging assessments with a mean time of 2.65 years (SD = 1.09; Range 1.00–7.34 years) between assessments.

### Associations between recent SLEs and brain structure

The estimates of the main effects of SLE group and time are reported in Table 2 for the LMMs fitted for each brain structure. Where the interaction term for SLE group × time was not significant, the reported coefficient estimates are from the models where we have excluded the interaction term.

**Table 2.** Fixed effect estimates from the linear mixed models comparing brain structure between participants who have or have not reported recent stressful life events

	SLE group^a^			Time (years from first imaging assessment)			SLE group × time^b^		
Brain structure (dependent variable)	b	95% CI	*p*-Value	b	95% CI	*p*-Value	b	95% CI	*p*-Value
Total gray matter volume	−0.277	[−0.903, 0.349]	0.386	−1.842	[−1.981, −1.704]	< 0.001**,^†^	–	–	–
Hippocampus volume	−0.010	[−0.023, 0.003]	0.142	−0.054	[−0.057, −0.051]	< 0.001**,^†^	0.007	[0.002, 0.013]	0.010*,^†^
Amygdala volume	0.004	[−0.001, 0.010]	0.133	−0.012	[−0.013, −0.011]	< 0.001**,^†^	–	–	–
Orbitofrontal cortical thickness	−0.002	[−0.004, 0.001]	0.165	−0.004	[−0.004, −0.003]	< 0.001**,^†^	–	–	–
Anterior cingulate cortical thickness	−0.001	[−0.004, 0.001]	0.297	−4.62 × 10^−4^	[−1.34 × 10^−4^, 0.001]	0.129	–	–	–
Insula cortical thickness	−0.002	[−0.004, 0.001]	0.250	−0.003	[−0.004, −0.003]	< 0.001**,^†^	–	–	–

Abbreviations: SLE, stressful life events.

The linear mixed models included participants (*n* = 4,543) modeled as random intercepts. All models controlled for sex, age at first imaging assessment, age^2^, total intracranial volume, and scan centre.

a Reference category for SLE group is SLE– (coded as 0) comprising individuals who have not reported a recent SLE.

b Where the interaction term of SLE group **×** time was not significant, the fixed effects from the model excluding the interaction term are reported. Only the hippocampus estimates are from the model that included the interaction term. Full results are present in Supplementary Table S3.

* *p* < 0.05.

** *p* < 0.01.

† Findings survived false discovery rate correction for multiple comparisons (*p*-values reported are uncorrected).

**Figure 1.**
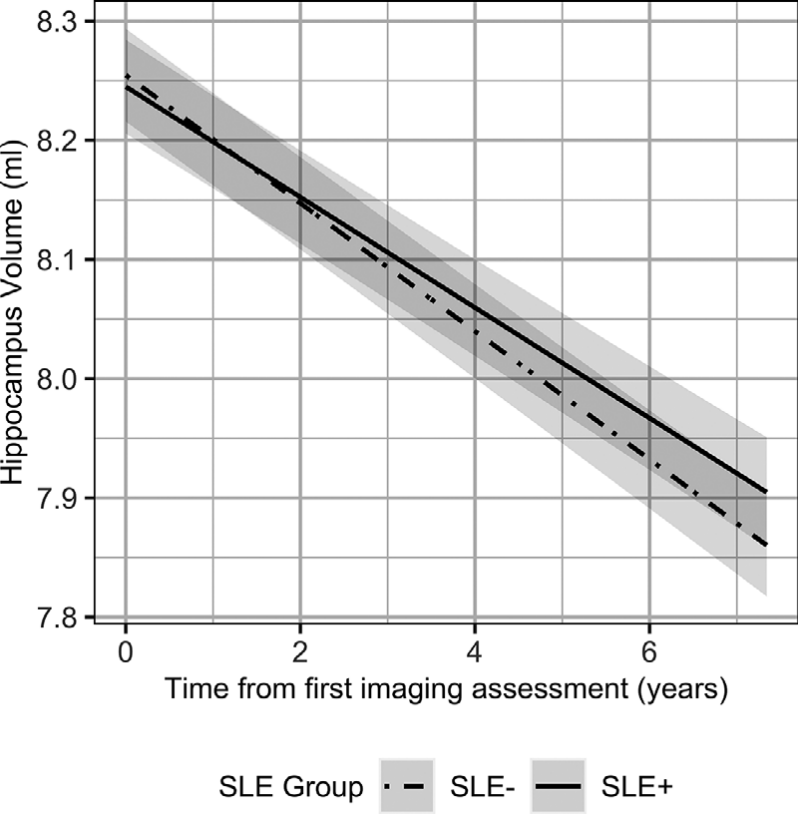
The predicted marginal effects from the linear mixed model showing different trajectories of the SLE− and SLE+ groups for hippocampal volume changes over the study follow-up period. The shaded regions represent the 95% confidence interval. Abbreviations: SLE, stressful life event; SLE−, individuals who reported no recent SLEs; SLE+, individuals who reported one or more recent SLE.

Only the hippocampus revealed a significant SLE group × time interaction, where hippocampal volumes decreased over time at a slower rate in the SLE+ group as compared to the SLE− group (seen by the different slopes in Figure 1). In all other brain regions, SLE group did not have a significant effect, suggesting there was no difference in brain structure between SLE+ and SLE− when controlling for time. All brain regions, except for the ACC, reported a significant effect of time, exhibiting a reduction in GM volumes and in mean cortical thickness over the follow-up period. The full model results are reported in Supplementary Table S3.

When we examined for associations between the SLE score and brain structure within the SLE+ group, we did not find any significant effect of the interaction term SLE score × time, nor of the SLE score as a main effect (see Supplementary Table S4). This suggests the number of events was not associated with brain structure.

### Exploratory analyses of the effects of depression and childhood adversity

**Figure 2.**
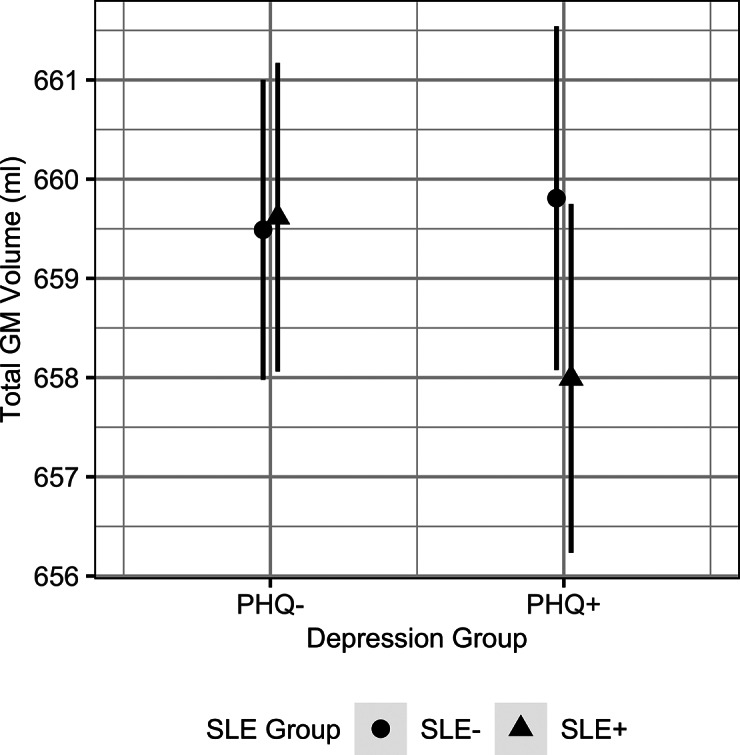
The predicted marginal effects from the linear mixed model showing the differences in total GM volumes between depression groups depending on recent SLE exposure (SLE group). The error bars represent the 95% confidence interval. Abbreviations: GM, gray matter; SLE, stressful life event; SLE−, individuals who reported no recent SLEs; SLE+, individuals who reported one or more recent SLE; PHQ+, individuals with probable depression; PHQ−, individuals without probable depression.

The results from the exploratory analysis investigating the effects of depression group (PHQ+ or PHQ–) are reported in Table 3. The interaction term SLE group × depression group only had a significant effect on total GM volume. Individuals with probable depression exhibited smaller total GM volumes where they reported recent SLEs, compared to those who did not report any recent SLEs (Figure 2). We also observed a significant interaction effect of SLE group × depression group on hippocampal volumes, however this did not pass correction (*p_corrected_* = 0.070). The full model estimates are reported in Supplementary Table S5.

**Table 3.** Fixed effect estimates from the linear mixed models examining the association between brain structure and recent stressful life events and depression

	SLE group^a^			Depression group^b^			SLE group × depression group^c^		
Brain structure (dependent variable)	b	95% CI	*p*-Value	b	95% CI	*p*-Value	b	95% CI	*p*-Value
Total gray matter volume	0.126	[−0.595, 0.848]	0.731	0.318	[−0.758, 1.395]	0.562	−1.943	[−3.370, −0.516]	0.008**^,^^†^
Hippocampus volume	−0.004	[−0.016, 0.008]	0.503	−0.018	[−0.036, −7.73 × 10^−5^]	0.049*	0.029	[0.005, 0.052]	0.018*
Amygdala volume	0.006	[8.02 × 10^−5^, 0.011]	0.047*	−0.001	[−0.009, 0.007]	0.795	–	–	–
Orbitofrontal cortical thickness	−0.001	[−0.004, 0.001]	0.274	0.001	[−0.002, 0.005]	0.402	–	–	–
Anterior cingulate cortical thickness	−0.001	[−0.004, 0.002]	0.397	0.004	[3.51 × 10^−4^, 0.008]	0.032*	–	–	–
Insula cortical thickness	−0.001	[−0.004, 0.002]	0.518	−0.001	[−0.005, 0.003]	0.648	–	–	–

Abbreviations: PHQ, Patient Health Questionnaire; SLE, stressful life events.

The linear mixed models included participants (*n* = 4,506) modeled as random intercepts. All models controlled for time (in years from first imaging assessment), sex, age at first imaging assessment, age^2^, total intracranial volume, and scan centre.

a Reference category for SLE group is SLE– (coded as 0), comprising individuals who have not reported a recent SLE.

b Reference category for the depression group is PHQ– (coded as 0) comprising individuals who are unlikely to have a depressive disorder based on the cut-off score (<3) for the PHQ-2.

c Where the interaction term of SLE group × depression group was not significant, the fixed effects from the model excluding the interaction term are reported. Only total GMV and the hippocampus estimates are from the model that included the interaction term. Full results are present in Supplementary Table S5.

* *p* < 0.05.

** *p* < 0.01.

† Findings survived false discovery rate correction for multiple comparisons (*p*-values reported are uncorrected).

We did not find a significant interaction between SLE group and CA group associated with brain structure, nor was there a main effect of CA group on brain structure in the subsequent models excluding the interaction term (all *p* > 0.05). Our results indicate there were no differences in brain structure between individuals who had experienced childhood adversity and those who had not. The full model estimates are reported in Supplementary Table S6.

### Sensitivity analyses

There were no changes to the significance of our results when we excluded individuals who reported having a stroke in their lifetime or outlier total ICV, nor when we included the lifestyle and sociodemographic variables as covariates of no interest. When we expanded our model to include the additional neuroimaging confounders, the interaction between SLE group × time associated with the hippocampus was no longer significant.

### Whole-brain exploratory analysis

The results of the whole-brain analysis are reported in the Supplementary Table S7 for group differences between SLE+ and SLE−. None of the findings survived correction for multiple comparisons.

## Discussion

We investigated the effects of recent SLEs on brain structure using a longitudinal neuroimaging dataset from a large population cohort. The hippocampus exhibited a slower decline in GM volume over the study period in individuals with recent SLE exposure compared to those without recent SLE exposure. In exploratory analysis, total GM volume differed between SLE exposure groups in individuals with depression but not in non-depressed individuals. We found childhood adversity had no effect on the relationship between recent SLEs and brain structure.

Our results exhibited a decrease in hippocampal volumes with time, which is expected in terms of aging-related changes [44]. However, contrary to our expectations, the SLE+ group exhibited a slower rate of volume reduction over the follow-up period. The hippocampus is highly plastic, and while this may make it a region of vulnerability in many disorders [45], hippocampal GM volume reduction might be countered through mental stimulation, exercise, or social interaction [46–50], which may serve as protective factors. This may explain our findings, as a higher proportion of the SLE+ group were still in employment, which could suggest higher mental stimulation, and were found to consume less alcohol, a risk factor associated with brain shrinkage [51, 52]. However, there were no changes to our results when we controlled for employment status and alcohol intake in a sensitivity analysis. The interaction between SLE group and time was no longer significant when we expanded our model to include a wider set of neuroimaging confounders. However, the difference in the rate of hippocampal volume change between SLE groups was very subtle, estimated to be slower by 0.007 ml/year in the SLE+ group (see the interaction term of SLE group × time in Table 2).

It is possible that stress may pose more of a risk at a younger age, given the global median age of onset for stress-related disorders was found to be below 35 years [53]. Neuroimaging studies investigating stress in older cohorts (>60 years) are limited. One longitudinal study, in a depressed and non-depressed sample (*N* = 159, mean age = 70 years), found that SLEs were associated with larger hippocampal volumes at baseline but there was no evidence of a temporal association between SLEs and brain structure [54]. Another cross-sectional study (*N* = 466, mean age = 71 years) reported that SLEs that occurred over the age of 65 were associated with greater amygdala volumes, but not with hippocampal volumes [55]. Previous longitudinal studies by Papagni et al. [14] and Ringwald et al. [15] that found significant associations between recent SLEs and changes in brain structure analyzed younger samples with mean ages of 25.2 years (*N* = 26) and 32.8 years (*N* = 212) respectively, suggesting stress-induced changes could be more prominent in younger populations.

Stress is subjective to an individual’s experience, and it may be the perception of stressful events that is more relevant to structural brain changes rather than the occurrence of an event. Previous cross-sectional work has reported associations between higher perceived stress levels and smaller GM volumes within the prefrontal cortex [56] and insula [17], and a longitudinal study has suggested that a smaller hippocampus represents a vulnerability to stress [57]. There is also some evidence of rumination being associated with larger GM volumes within the prefrontal cortex and ACC [58], which may have affected our results. As such, future studies could incorporate subjective measures of stress and rumination.

We found total GM volume differed between depressed individuals with and without recent SLE exposure, but not in nondepressed individuals. Stress is linked to the onset of depression [36, 37] and severe subclinical depressive symptoms have been associated with smaller GM volumes [59, 60]. Our findings indicate that recent stress may influence the association between depressive symptoms and total GM volume as has been previously reported [61]. However, further work is required to clarify the direction of effect as smaller GM volumes have been associated with major depressive disorder in non-stress studies [62, 63]. In addition, affective-disorder psychopathology could result in an individual becoming susceptible to SLEs [64, 65], subsequently leading to further harmful effects. We did not observe any effects of childhood adversity on the association between recent SLEs and brain structure. However, as childhood adversity is thought to increase sensitivity to stress [40, 41], perceived stress levels may be more relevant in this context.

The current study had several limitations. Firstly, the questionnaire capturing recent SLEs was limited to six events. While these events are found in other validated life event questionnaires [66, 67], they did not capture other event types such as having serious problems with a friend or being the victim of theft. The questionnaire also did not facilitate the reporting of multiple events of the same type, meaning the data may have underreported the number of SLEs. The UK Biobank data comprised individuals from mainly white European ethnic backgrounds (97% of the current sample), and older adults, affecting the generalizability of results to other racial and ethnic groups. In addition, we may be observing a survival bias in the study sample as participants have actively participated in repeated data collection, which could indicate that they are overall healthier and perhaps more resilient to stress. The time between assessments varied with some participants completing a follow-up assessment more than 2 years after their first imaging assessment. It is, therefore, possible that SLEs with potentially impactful or lasting effects may have occurred outside of the 2-year period defining a recent SLE, and were therefore not accounted for in the study. Future study design using experience sampling methods to record daily stressors over a shorter period may be an alternative approach to capture an individual’s experience of stress.

In conclusion, using longitudinal neuroimaging data from a large population cohort, we have found that recent SLEs may not accelerate brain structure reductions in older adults, but may influence changes through affective disorder psychopathology. Further research is needed to uncover the effects of stress on the general population, with a particular focus on younger populations, who may be more vulnerable to stress-induced changes. This work may yet pinpoint vulnerable brain regions linked to stress-related disorders.

## Supporting information

See et al. supplementary material See et al. supplementary material

## Data Availability

Data are available via the UK Biobank (https://www.ukbiobank.ac.uk).

## Supplementary material

The supplementary material for this article can be found at https://doi.org/10.1192/j.eurpsy.2025.2.
