# Supplementary material for: The association between recent stressful life events and brain structure: a UK Biobank longitudinal MRI study
Source: Eur Psychiatry. 2025 Jan 21;68(1):e18. doi: 10.1192/j.eurpsy.2025.2 (PMC11822962; doi:10.1192/j.eurpsy.2025.2)
Supplement: See et al. supplementary material [file S0924933825000021sup001.docx]

# Supplementary Material

[**Figure S1.** Timeline of data collection for the UK Biobank. 2](#_Toc181946055)

[**Figure S2.** Flow chart of the sample sizes used in the analyses. 2](#_Toc181946056)

[**Table S1.** List of UK Biobank variables used in the study. 3](#_Toc181946057)

[**Supplementary Methods** 4](#_Toc181946058)

[**Table S2.** Frequencies of reported stressful life events at the first imaging assessment in the SLE+ group (*n* = 1,749). 6](#_Toc181946059)

[**Table S3.** Model estimates from the linear mixed models investigating the association between SLE group and brain structure. 7](#_Toc181946060)

[**Table S4.** Model estimates from the linear mixed models investigating the association between SLE score and brain structure in participants who reported one or more SLEs. 9](#_Toc181946061)

[**Table S5.** Model estimates from the exploratory analysis investigating for the effects of depression. 11](#_Toc181946062)

[**Table S6.** Model estimates from the exploratory analysis investigating for the effects of childhood adversity. 13](#_Toc181946063)

[**Table S7.** SLE group estimates across all brain structures. 15](#_Toc181946064)

[**Supplementary References** 18](#_Toc181946065)

*Notes:*

- The current study uses participant data from the **first and repeat imaging assessments** (including the SLE questionnaire and depression symptom data), except for variables that were only recorded at the baseline assessment (2006) including sex and the Townsend deprivation index.
- All variables used in the analysis were obtained from the main in-person assessments except for childhood adversity data which were obtained from the Mental Health questionnaire, a one-off on-line assessment conducted in 2016.

Figure S1. Timeline of data collection for the UK Biobank.

Notes: SLE = stressful life event; *SLE- = individuals who reported no recent SLEs; SLE+ = individuals who reported one or more recent SLE;* QC = quality control. PHQ-2 = Patient Health Questionnaire-4 depression subscale; CTS-5 = Childhood Trauma Screener.

N represents the number of unique participants (not the number of observations) in each data subset with data at the first and repeat imaging assessment (two timepoints).

Figure S2. Flow chart of the sample sizes used in the analyses.

Table S1. List of UK Biobank variables used in the study.

| **Data-Field ID** | **Field Description** |
| --- | --- |
| 6145 | Illness, injury, bereavement, stress in last 2 years |
| 21003 | Age when attended assessment centre |
| 31 | Sex |
| 53 | Date of attending assessment centre |
| 54 | UK Biobank assessment centre |
| 20489 | Felt loved as a child |
| 20488 | Physically abused by family as a child |
| 20487 | Felt hated by family member as a child |
| 20490 | Sexually molested as a child |
| 20491 | Someone to take to doctor when needed as a child |
| 2050 | Frequency of depressed mood in last 2 weeks |
| 2060 | Frequency of unenthusiasm/disinterest in last 2 weeks |
| 6142 | Current employment status |
| 2188 | Long-standing illness, disability or infirmity |
| 1558 | Alcohol intake frequency |
| 20116 | Smoking status |
| 22189 | Townsend Deprivation Index |
| 6150 | Vascular/heart problems diagnosed by doctor |
| 25741 | Mean resting fMRI head motion, averaged across space and time points |
| 25742 | Mean task fMRI head motion, averaged across space and time points |

*Details relating to each variable can be accessed via the UK Biobank data showcase:* [*https://biobank.ndph.ox.ac.uk/showcase/index.cgi*](https://biobank.ndph.ox.ac.uk/showcase/index.cgi)

Supplementary Methods

***Quality control of imaging outputs***

The FreeSurfer output were quality controlled following the ENIGMA Quality Control (QC) Protocol (<https://enigma.ini.usc.edu/protocols/imaging-protocols>). Summary statistics were generated for the total intracranial volume (ICV), total grey matter (GM) volume, and for each region of interest in the current study: bilateral subcortical GM volumes of the hippocampus and amygdala, and the mean cortical thicknesses of the orbitofrontal cortex (OFC), anterior cingulate cortex (ACC), and insula. Outliers were identified as being 2.698 times the standard deviation above or below the mean of a structural measure, based on the ENIGMA QC protocol. This value was based on the interquartile interval, which is 1.5 times the interquartile range (IQR) below Quartile 1, to 1.5 times the IQR above Quartile 3, which is equivalent to 2.698 times the standard deviation above and below the mean in a normal distribution. The outlier participant scans were visually inspected for any issues with the segmentation process during the FreeSurfer analysis. As a result, seven participants were excluded due to poor image quality of the original scan affecting segmentation.

***Treatment of Variables***

Below provides additional details regarding the treatment of covariates. Across all fields, where participants responded with either “Prefer not to answer” or “Do not know”, these responses were treated as missing data and excluded from the relevant analysis. Variables with multiple categories were dummy coded to use in the regression models. Table S1 contains a full list of the variables used in the current study including their corresponding UKB Data-Field identification numbers.

**Depression group.** The responses from two items in the UKB ([Data-Fields 2050 and 2060](https://biobank.ndph.ox.ac.uk/showcase/label.cgi?id=100060)), make up the Patient Health Questionnaire (PHQ)-4 depression subscale [1, 2]. These items were responded to using a scale ranging from 1 = “Not at all” to 4 = ”Nearly every day”. Prior to summing the scores across the two items to calculate a PHQ-4 depression subscale score, we subtracted 1 from each item such that the response for “Not at all” equalled a score of zero. This translated the scores such that a total score of zero now corresponded to a participant reporting no recent depressive symptoms. The scores were then used to group participants into PHQ+ (probable depression) or PHQ- (nondepressed), as described in the main manuscript.

**Current employment status.** The responses were consolidated to reduce the number of categories from eight to five where: 1 = In employment; 2 = Retired; 3 = Unable to work due to disability or sickness/looking after home and/or family /student/volunteer or unpaid work; 4 = Unemployed; 5 = Other (None of the above). This was to combine subcategories that were similar in nature with low frequency counts (<1% of the study sample). The variable was dummy coded with the reference category as “1 = In employment”.

**Long-standing illness, disability or infirmity.** Participants responded as either “Yes” or “No” to this question, which we treated as a dichotomous variable. Responses for “Do not know” were treated as missing data.

**Alcohol intake frequency.** The responses were consolidated to recategorize alcohol intake frequency from six to four categories of 0 = never, 1 = rarely/monthly, 2 = weekly, and 3 = daily. Participant responses for “Special occasions only” and “1-3 times a month” were grouped together as “1 = rarely/monthly”. Responses of “1-2 times a week” or “3-4 times a week” were grouped together as “2 = weekly”. Responses for “Daily” or “Almost daily” were grouped together as “3 = daily”. The variable was dummy coded with the reference category set as “0 = Never”.

**Townsend deprivation index.** The data were used as is from the UK Biobank and was measured at the time of recruitment. The Townsend deprivation index is a score accounting for unemployment, overcrowding, non-car, and non-home ownership, and was determined by a participant’s area of residence at recruitment [3]. A higher score indicates higher levels of socioeconomic deprivation.

**Drift Time.** This is the time between the first scan conducted at a given imaging centre, and a participant’s actual scan date. Drift time is a suggested proxy for capturing the effect caused by a scanners’ resonance frequency drift [4, 5]. This was derived using the earliest imaging assessment date ([Data-Field 53](https://biobank.ndph.ox.ac.uk/showcase/field.cgi?id=53)) for each centre. The dates were subtracted to measure the time in days, and subsequently divided by 365 to convert it to years.

***Sensitivity analyses including neuroimaging confounders***

As sensitivity analyses, we re-fit the main models with SLE group as a fixed effect and included a broader range of neuroimaging confounders as identified by Alfaro-Almagro et al. [5]. This included age (at first imaging assessment), age^2^, sex, total ICV, age x sex, age^2^ x sex, drift time, drift time^2^, mean resting-state functional MRI head motion, mean task-based functional MRI head motion, time (years from first imaging assessment), time^2^, and centre.

Table S2. Frequencies of reported stressful life events at the first imaging assessment in the SLE+ group (*n* = 1,749).

| **Stressful Event** | **Frequency (%)** |
| --- | --- |
| Serious illness, injury or assault to yourself | 237 (13.6) |
| Serious illness, injury or assault of a close relative | 630 (36.0) |
| Death of a close relative | 927 (53.0) |
| Death of a spouse or partner | 49 (2.8) |
| Marital separation/divorce | 85 (4.9) |
| Financial difficulties | 279 (16.0) |

Note: As participants could only report a single occurrence of each event, frequency represents the number of participants who reported each event. The percentage is therefore calculated as a proportion of participants who reported at least one stressful life event at the first imaging assessment.

Table S3. Model estimates from the linear mixed models investigating the association between SLE group and brain structure.

| **Brain Structure**  **(Dependent Variable)** |  |  |  |  |  |  |  |  | ***95% CI*** | |  |
| --- | --- | --- | --- | --- | --- | --- | --- | --- | --- | --- | --- |
|  | **Model Fit** |  | **Random Effects** |  | **Fixed Effects** | ***Estimate*** | ***SE*** | ***t*** | ***Lower*** | ***Upper*** | ***P*** |
| Total GM volume (ml) | R^2^ (Conditional) | 0.970 | Participants, SD | 23.99 | (Intercept) | 661.699 | 0.766 | 864.013 | 660.198 | 663.200 | < 1×10^-300^ ** |
|  | R^2^ (Marginal) | 0.783 | ICC, % | 86.1 | **SLE Group** | -0.277 | 0.319 | -0.866 | -0.903 | 0.349 | .386 |
|  |  |  |  |  | **Time** | -1.842 | 0.070 | -26.146 | -1.981 | -1.704 | 1.19×10^-140^ **^,a^ |
|  |  |  |  |  | Age | -11.004 | 0.381 | -28.898 | -11.751 | -10.258 | 9.05×10^-169^ ** |
|  |  |  |  |  | Age^2^ | -2.160 | 0.354 | -6.106 | -2.854 | -1.467 | 1.11×10^-9^ ** |
|  |  |  |  |  | Sex | 11.840 | 0.895 | 13.230 | 10.086 | 13.594 | 3.11×10^-39^ ** |
|  |  |  |  |  | Total ICV | 43.993 | 0.445 | 98.850 | 43.121 | 44.866 | < 1×10^-300^ |
|  |  |  |  |  | Centre - Reading | -5.072 | 1.327 | -3.823 | -7.672 | -2.471 | 1.34×10^-4^ ** |
|  |  |  |  |  | Centre - Newcastle | -1.383 | 0.802 | -1.723 | -2.955 | 0.190 | .085 |
| Hippocampus volume (ml) | R^2^ (Conditional) | 0.965 | Participants, SD | 0.64 | (Intercept) | 8.255 | 0.020 | 415.896 | 8.216 | 8.294 | < 1×10^-300^ ** |
|  | R^2^ (Marginal) | 0.404 | ICC, % | 94.1 | **SLE Group** | -0.010 | 0.007 | -1.470 | -0.023 | 0.003 | .142 |
|  |  |  |  |  | **Time** | -0.054 | 0.002 | -33.648 | -0.057 | -0.051 | 2.43×10^-222^ **^,a^ |
|  |  |  |  |  | **SLE Group x Time** | 0.007 | 0.003 | 2.575 | 0.002 | 0.013 | .010*^,a^ |
|  |  |  |  |  | Age | -0.350 | 0.010 | -35.105 | -0.369 | -0.330 | 4.13×10^-239^ ** |
|  |  |  |  |  | Age^2^ | -0.051 | 0.009 | -5.554 | -0.070 | -0.033 | 2.95×10^-8^ ** |
|  |  |  |  |  | Sex | 0.083 | 0.023 | 3.545 | 0.037 | 0.129 | 3.96×10^-4^ ** |
|  |  |  |  |  | Total ICV | 0.379 | 0.012 | 32.514 | 0.356 | 0.402 | 1.22×10^-208^ ** |
|  |  |  |  |  | Centre - Reading | -0.090 | 0.035 | -2.606 | -0.159 | -0.022 | .009* |
|  |  |  |  |  | Centre - Newcastle | 0.001 | 0.021 | 0.065 | -0.040 | 0.043 | .949 |
| Amygdala volume (ml) | R^2^ (Conditional) | 0.963 | Participants, SD | 0.31 | (Intercept) | 3.284 | 0.010 | 341.896 | 3.265 | 3.303 | < 1×10^-300^ ** |
|  | R^2^ (Marginal) | 0.429 | ICC, % | 93.5 | **SLE Group** | 0.004 | 0.003 | 1.503 | -0.001 | 0.010 | .133 |
|  |  |  |  |  | **Time** | -0.012 | 0.001 | -20.367 | -0.013 | -0.011 | 2.45×10^-88^ **^,a^ |
|  |  |  |  |  | Age | -0.125 | 0.005 | -25.846 | -0.134 | -0.115 | 1.48×10^-137^ ** |
|  |  |  |  |  | Age^2^ | -0.024 | 0.004 | -5.328 | -0.033 | -0.015 | 1.04×10^-7^ ** |
|  |  |  |  |  | Sex | 0.134 | 0.011 | 11.755 | 0.111 | 0.156 | 1.89×10^-31^ ** |
|  |  |  |  |  | Total ICV | 0.203 | 0.006 | 35.957 | 0.192 | 0.214 | 2.43×10^-249^ ** |
|  |  |  |  |  | Centre - Reading | -0.045 | 0.017 | -2.659 | -0.078 | -0.012 | .008* |
|  |  |  |  |  | Centre - Newcastle | -0.012 | 0.010 | -1.220 | -0.032 | 0.008 | .223 |

**Table S3 *(continued)*.** Model estimates from the linear mixed models investigating the association between SLE group and brain structure.

| **Brain Structure**  **(Dependent Variable)** |  |  |  |  |  |  |  |  | ***95% CI*** | |  |
| --- | --- | --- | --- | --- | --- | --- | --- | --- | --- | --- | --- |
|  | **Model Fit** |  | **Random Effects** |  | **Fixed Effects** | ***Estimate*** | ***SE*** | ***t*** | ***Lower*** | ***Upper*** | ***P*** |
| Orbitofrontal cortical thickness (mm) | R^2^ (Conditional) | 0.814 | Participants, SD | 0.08 | (Intercept) | 2.484 | 0.003 | 972.798 | 2.479 | 2.489 | < 1×10^-300^ ** |
|  | R^2^ (Marginal) | 0.042 | ICC, % | 80.5 | **SLE Group** | -0.002 | 0.001 | -1.388 | -0.004 | 0.001 | .165 |
|  |  |  |  |  | **Time** | -0.004 | 2.79×10^-4^ | -13.234 | -0.004 | -0.003 | 2.85×10^-39^ **^,a^ |
|  |  |  |  |  | Age | -0.015 | 0.001 | -11.549 | -0.017 | -0.012 | 1.98×10^-30^ ** |
|  |  |  |  |  | Age^2^ | 0.001 | 0.001 | 0.797 | -0.001 | 0.003 | .425 |
|  |  |  |  |  | Sex | 0.024 | 0.003 | 8.203 | 0.018 | 0.030 | 3.02×10^-16^ ** |
|  |  |  |  |  | Total ICV | -0.007 | 0.001 | -4.422 | -0.009 | -0.004 | 9.99×10^-6^ ** |
|  |  |  |  |  | Centre - Reading | -0.006 | 0.004 | -1.294 | -0.014 | 0.003 | .196 |
|  |  |  |  |  | Centre - Newcastle | -0.013 | 0.003 | -5.026 | -0.019 | -0.008 | 5.21×10^-7^ ** |
| Anterior cingulate cortical thickness (mm) | R^2^ (Conditional) | 0.892 | Participants, SD | 0.12 | (Intercept) | 2.528 | 0.004 | 693.549 | 2.521 | 2.535 | < 1×10^-300^ ** |
|  | R^2^ (Marginal) | 0.061 | ICC, % | 88.5 | **SLE Group** | -0.001 | 0.001 | -1.043 | -0.004 | 0.001 | .297 |
|  |  |  |  |  | **Time** | 4.62×10^-4^ | 3.04×10^-4^ | 1.519 | -1.34×10^-4^ | 0.001 | .129 |
|  |  |  |  |  | Age | 0.009 | 0.002 | 4.893 | 0.005 | 0.012 | 1.03×10^-6^ ** |
|  |  |  |  |  | Age^2^ | 0.007 | 0.002 | 4.170 | 0.004 | 0.010 | 3.10×10^-5^ ** |
|  |  |  |  |  | Sex | 0.014 | 0.004 | 3.169 | 0.005 | 0.022 | .002** |
|  |  |  |  |  | Total ICV | -0.031 | 0.002 | -14.568 | -0.035 | -0.027 | 5.12×10^-47^ ** |
|  |  |  |  |  | Centre - Reading | -0.015 | 0.006 | -2.288 | -0.027 | -0.002 | .022* |
|  |  |  |  |  | Centre - Newcastle | -0.015 | 0.004 | -3.820 | -0.022 | -0.007 | 1.35×10^-4^ ** |
| Insula cortical thickness (mm) | R^2^ (Conditional) | 0.876 | Participants, SD | 0.11 | (Intercept) | 2.861 | 0.003 | 830.879 | 2.854 | 2.868 | < 1×10^-300^ ** |
|  | R^2^ (Marginal) | 0.012 | ICC, % | 87.4 | **SLE Group** | -0.002 | 0.001 | -1.149 | -0.004 | 0.001 | .250 |
|  |  |  |  |  | **Time** | -0.003 | 3.01×10^-4^ | -10.499 | -0.004 | -0.003 | 1.68×10^-25^ ** |
|  |  |  |  |  | Age | -0.009 | 0.002 | -5.218 | -0.012 | -0.006 | 1.89×10^-7^ ** |
|  |  |  |  |  | Age^2^ | 0.001 | 0.002 | 0.735 | -0.002 | 0.004 | .462 |
|  |  |  |  |  | Sex | 0.016 | 0.004 | 3.973 | 0.008 | 0.024 | 7.22×10^-5^ ** |
|  |  |  |  |  | Total ICV | -0.002 | 0.002 | -0.833 | -0.006 | 0.002 | .405 |
|  |  |  |  |  | Centre - Reading | 0.007 | 0.006 | 1.102 | -0.005 | 0.018 | .271 |
|  |  |  |  |  | Centre - Newcastle | -0.009 | 0.004 | -2.400 | -0.016 | -0.002 | .016* |

*p < .05; **p < .01. The p-values reported above are uncorrected.

^a^ Variables of interest (highlighted rows) survived false discovery rate (FDR) correction for multiple comparisons.

*Note:* Where the interaction term of SLE Group x Time was not significant, the fixed effects from the model excluding the interaction term are reported above. Only the hippocampus estimates are from the model that included the interaction term.

The linear mixed models included participants (*n* = 4,543) modelled as random intercepts. Time is measured in years from the first imaging assessment. All models controlled for sex, age at first imaging assessment, age^2^, total intracranial volume, and scan centre. Age and total ICV were standardised and mean centred to avoid large variation in scales across covariates affecting model convergence.

SLE = stressful life event; ICV = intracranial volume; ICC = intraclass correlation coefficient.

Categorical Variables: SLE Group: 0 = SLE-, 1 = SLE+; Sex: 0 = Female, 1 = Male; Centre: 0 = Cheadle, 1 = Reading, 2 = Newcastle.

Table S4. Model estimates from the linear mixed models investigating the association between SLE score and brain structure in participants who reported one or more SLEs.

| **Brain Structure**  **(Dependent Variable)** |  |  |  |  |  |  |  |  | ***95% CI*** | |  |
| --- | --- | --- | --- | --- | --- | --- | --- | --- | --- | --- | --- |
|  | **Model Fit** |  | **Random Effects** |  | **Fixed Effects** | ***Estimate*** | ***SE*** | ***t*** | ***Lower*** | ***Upper*** | ***P*** |
| Total GM volume (ml) | R^2^ (Conditional) | 0.971 | Participants, SD | 23.99 | (Intercept) | 662.850 | 1.271 | 521.369 | 660.358 | 665.341 | < 1×10^-300^ ** |
|  | R^2^ (Marginal) | 0.790 | ICC, % | 86.4 | **SLE Score** | -0.376 | 0.560 | -0.671 | -1.474 | 0.722 | .503 |
|  |  |  |  |  | **Time** | -1.984 | 0.147 | -13.522 | -2.271 | -1.696 | 7.31×10^-39^ **^,a^ |
|  |  |  |  |  | Age | -11.070 | 0.533 | -20.760 | -12.115 | -10.025 | 1.47×10^-88^ ** |
|  |  |  |  |  | Age^2^ | -2.352 | 0.502 | -4.683 | -3.336 | -1.368 | 2.97×10^-6^ ** |
|  |  |  |  |  | Sex | 10.112 | 1.229 | 8.227 | 7.703 | 12.521 | 3.03×10^-16^ ** |
|  |  |  |  |  | Total ICV | 45.243 | 0.611 | 74.041 | 44.045 | 46.441 | < 1×10^-300^ ** |
|  |  |  |  |  | Centre - Reading | -5.657 | 1.882 | -3.005 | -9.347 | -1.968 | .003** |
|  |  |  |  |  | Centre - Newcastle | -1.611 | 1.104 | -1.459 | -3.776 | 0.554 | .145 |
| Hippocampus volume (ml) | R^2^ (Conditional) | 0.969 | Participants, SD | 0.64 | (Intercept) | 8.256 | 0.029 | 282.716 | 8.199 | 8.313 | < 1×10^-300^ ** |
|  | R^2^ (Marginal) | 0.410 | ICC, % | 94.7 | **SLE Score** | 0.017 | 0.010 | 1.790 | -0.002 | 0.036 | .074 |
|  |  |  |  |  | **Time** | -0.045 | 0.002 | -18.654 | -0.050 | -0.040 | 7.19×10^-67^ **^,a^ |
|  |  |  |  |  | Age | -0.339 | 0.014 | -25.005 | -0.366 | -0.313 | 1.75×10^-123^ ** |
|  |  |  |  |  | Age^2^ | -0.059 | 0.013 | -4.636 | -0.084 | -0.034 | 3.74×10^-6^ ** |
|  |  |  |  |  | Sex | 0.052 | 0.031 | 1.659 | -0.009 | 0.113 | .097 |
|  |  |  |  |  | Total ICV | 0.389 | 0.016 | 24.973 | 0.359 | 0.420 | 3.75×10^-123^ ** |
|  |  |  |  |  | Centre - Reading | -0.090 | 0.048 | -1.877 | -0.184 | 0.004 | .061 |
|  |  |  |  |  | Centre - Newcastle | -0.041 | 0.028 | -1.447 | -0.096 | 0.014 | .148 |
| Amygdala volume (ml) | R^2^ (Conditional) | 0.964 | Participants, SD | 0.31 | (Intercept) | 3.307 | 0.014 | 228.353 | 3.278 | 3.335 | < 1×10^-300^ ** |
|  | R^2^ (Marginal) | 0.453 | ICC, % | 93.4 | **SLE Score** | -0.005 | 0.005 | -0.909 | -0.015 | 0.005 | .364 |
|  |  |  |  |  | **Time** | -0.012 | 0.001 | -8.863 | -0.014 | -0.009 | 3.35×10^-18^ **^,a^ |
|  |  |  |  |  | Age | -0.127 | 0.007 | -19.281 | -0.140 | -0.114 | 2.12×10^-77^ ** |
|  |  |  |  |  | Age^2^ | -0.036 | 0.006 | -5.714 | -0.048 | -0.023 | 1.24×10^-8^ ** |
|  |  |  |  |  | Sex | 0.137 | 0.015 | 8.984 | 0.107 | 0.167 | 4.98×10^-19^ ** |
|  |  |  |  |  | Total ICV | 0.211 | 0.008 | 27.829 | 0.196 | 0.226 | 7.58×10^-149^ ** |
|  |  |  |  |  | Centre - Reading | -0.045 | 0.023 | -1.939 | -0.091 | 4.93×10^-4^ | .053 |
|  |  |  |  |  | Centre - Newcastle | -0.021 | 0.014 | -1.522 | -0.048 | 0.006 | .128 |

**Table S4 *(continued)*.** Model estimates from the linear mixed models investigating the association between SLE score and brain structure in participants who reported one or more SLEs***.***

| **Brain Structure**  **(Dependent Variable)** |  |  |  |  |  |  |  |  | ***95% CI*** | |  |
| --- | --- | --- | --- | --- | --- | --- | --- | --- | --- | --- | --- |
|  | **Model Fit** |  | **Random Effects** |  | **Fixed Effects** | ***Estimate*** | ***SE*** | ***t*** | ***Lower*** | ***Upper*** | ***P*** |
| Orbitofrontal cortical thickness (mm) | R^2^ (Conditional) | 0.825 | Participants, SD | 0.08 | (Intercept) | 2.488 | 0.004 | 558.520 | 2.479 | 2.497 | < 1×10^-300^ ** |
|  | R^2^ (Marginal) | 0.043 | ICC, % | 81.7 | **SLE Score** | -0.004 | 0.002 | -1.740 | -0.008 | 4.64×10^-4^ | .082 |
|  |  |  |  |  | **Time** | -0.003 | 0.001 | -6.198 | -0.005 | -0.002 | 7.69×10^-10^ **^,a^ |
|  |  |  |  |  | Age | -0.014 | 0.002 | -7.997 | -0.018 | -0.011 | 1.91×10^-15^ ** |
|  |  |  |  |  | Age^2^ | 0.002 | 0.002 | 0.980 | -0.002 | 0.005 | .327 |
|  |  |  |  |  | Sex | 0.024 | 0.004 | 5.719 | 0.016 | 0.032 | 1.20×10^-8^ ** |
|  |  |  |  |  | Total ICV | -0.005 | 0.002 | -2.546 | -0.009 | -0.001 | .011** |
|  |  |  |  |  | Centre - Reading | -0.011 | 0.006 | -1.659 | -0.023 | 0.002 | .097 |
|  |  |  |  |  | Centre - Newcastle | -0.015 | 0.004 | -4.152 | -0.023 | -0.008 | 3.41×10^-5^ ** |
| Anterior cingulate cortical thickness (mm) | R^2^ (Conditional) | 0.899 | Participants, SD | 0.12 | (Intercept) | 2.521 | 0.006 | 430.181 | 2.510 | 2.533 | < 1×10^-300^ ** |
|  | R^2^ (Marginal) | 0.063 | ICC, % | 89.2 | **SLE Score** | 0.002 | 0.002 | 0.880 | -0.003 | 0.007 | .379 |
|  |  |  |  |  | **Time** | 2.72×10^-4^ | 0.001 | 0.434 | -0.001 | 0.002 | .664 |
|  |  |  |  |  | Age | 0.010 | 0.003 | 3.838 | 0.005 | 0.015 | 1.27×10^-4^ ** |
|  |  |  |  |  | Age^2^ | 0.012 | 0.002 | 4.832 | 0.007 | 0.016 | 1.43×10^-6^ ** |
|  |  |  |  |  | Sex | 0.010 | 0.006 | 1.654 | -0.002 | 0.021 | .098 |
|  |  |  |  |  | Total ICV | -0.029 | 0.003 | -10.075 | -0.035 | -0.024 | 1.98×10^-23^ ** |
|  |  |  |  |  | Centre - Reading | -0.011 | 0.009 | -1.256 | -0.029 | 0.006 | .209 |
|  |  |  |  |  | Centre - Newcastle | -0.013 | 0.005 | -2.418 | -0.023 | -0.002 | .016* |
| Insula cortical thickness (mm) | R^2^ (Conditional) | 0.876 | Participants, SD | 0.11 | (Intercept) | 2.856 | 0.006 | 510.187 | 2.845 | 2.867 | < 1×10^-300^ ** |
|  | R^2^ (Marginal) | 0.015 | ICC, % | 87.5 | **SLE Score** | 0.002 | 0.002 | 0.658 | -0.003 | 0.006 | .511 |
|  |  |  |  |  | **Time** | -0.002 | 0.001 | -3.635 | -0.004 | -0.001 | 2.90×10^-4^ **^,a^ |
|  |  |  |  |  | Age | -0.008 | 0.002 | -3.256 | -0.012 | -0.003 | .001** |
|  |  |  |  |  | Age^2^ | 0.001 | 0.002 | 0.562 | -0.003 | 0.006 | .574 |
|  |  |  |  |  | Sex | 0.019 | 0.005 | 3.525 | 0.009 | 0.030 | 4.30×10^-4^ ** |
|  |  |  |  |  | Total ICV | -0.001 | 0.003 | -0.385 | -0.006 | 0.004 | .700 |
|  |  |  |  |  | Centre - Reading | 0.006 | 0.008 | 0.677 | -0.011 | 0.022 | .498 |
|  |  |  |  |  | Centre - Newcastle | -0.015 | 0.005 | -3.030 | -0.025 | -0.005 | .002** |

*p < .05; **p < .01. The p-values reported above are uncorrected.

^a^ Variables of interest (highlighted rows) survived false discovery rate (FDR) correction for multiple comparisons.

*Note:* As the interaction term of SLE Score x Time was not significant, the fixed effects from the model excluding the interaction term are reported above.

The linear mixed models included participants with at least one SLE (*n* = 2,525) modelled as random intercepts. Time is measured in years from the first imaging assessment. All models controlled for sex, age at first imaging assessment, age^2^, total intracranial volume, and scan centre. Age and total ICV were standardised and mean centred to avoid large variation in scales across covariates affecting model convergence.

SLE = stressful life event; ICV = intracranial volume; ICC = intraclass correlation coefficient.

Categorical Variables: SLE Group: 0 = SLE-, 1 = SLE+; Sex: 0 = Female, 1 = Male; Centre: 0 = Cheadle, 1 = Reading, 2 = Newcastle.

Table S5. Model estimates from the exploratory analysis investigating for the effects of depression.

| **Brain Structure**  **(Dependent Variable)** |  |  |  |  |  |  |  |  | ***95% CI*** | |  |
| --- | --- | --- | --- | --- | --- | --- | --- | --- | --- | --- | --- |
|  | **Model Fit** |  | **Random Effects** |  | **Fixed Effects** | ***Estimate*** | ***SE*** | ***t*** | ***Lower*** | ***Upper*** | ***P*** |
| Total GM volume (ml) | R^2^ (Conditional) | 0.970 | Participants, SD | 23.9 | (Intercept) | 661.670 | 0.777 | 851.122 | 660.147 | 663.194 | < 1×10^-300^ ** |
|  | R^2^ (Marginal) | 0.784 | ICC, % | 86.1 | **SLE Group** | 0.126 | 0.368 | 0.343 | -0.595 | 0.848 | .731 |
|  |  |  |  |  | **Depression Group** | 0.318 | 0.549 | 0.580 | -0.758 | 1.395 | .562 |
|  |  |  |  |  | **SLE Group x Depression Group** | -1.943 | 0.728 | -2.669 | -3.370 | -0.516 | .008**^,a^ |
|  |  |  |  |  | Time | -1.832 | 0.072 | -25.396 | -1.974 | -1.691 | 6.88×10^-133^ ** |
|  |  |  |  |  | Age | -11.013 | 0.383 | -28.749 | -11.764 | -10.262 | 3.62×10^-167^ ** |
|  |  |  |  |  | Age^2^ | -2.164 | 0.356 | -6.079 | -2.862 | -1.467 | 1.31×10^-9^ ** |
|  |  |  |  |  | Sex | 11.785 | 0.899 | 13.109 | 10.023 | 13.547 | 1.47×10^-38^ ** |
|  |  |  |  |  | Total ICV | 44.018 | 0.447 | 98.517 | 43.142 | 44.893 | < 1×10^-300^ |
|  |  |  |  |  | Centre - Reading | -4.900 | 1.330 | -3.684 | -7.507 | -2.293 | 2.32×10^-4^ ** |
|  |  |  |  |  | Centre - Newcastle | -1.467 | 0.805 | -1.822 | -3.046 | 0.112 | .069 |
| Hippocampus volume (ml) | R^2^ (Conditional) | 0.967 | Participants, SD | 0.64 | (Intercept) | 8.252 | 0.020 | 412.388 | 8.213 | 8.292 | < 1×10^-300^ ** |
|  | R^2^ (Marginal) | 0.406 | ICC, % | 94.5 | **SLE Group** | -0.004 | 0.006 | -0.670 | -0.016 | 0.008 | .503 |
|  |  |  |  |  | **Depression Group** | -0.018 | 0.009 | -1.968 | -0.036 | -7.73×10^-5^ | .049* |
|  |  |  |  |  | **SLE Group x Depression Group** | 0.029 | 0.012 | 2.371 | 0.005 | 0.052 | .018* |
|  |  |  |  |  | Time | -0.050 | 0.001 | -43.303 | -0.053 | -0.048 | < 1×10^-300^ ** |
|  |  |  |  |  | Age | -0.350 | 0.010 | -34.859 | -0.369 | -0.330 | 5.32×10^-236^ ** |
|  |  |  |  |  | Age^2^ | -0.051 | 0.009 | -5.417 | -0.069 | -0.032 | 6.38×10^-8^ ** |
|  |  |  |  |  | Sex | 0.084 | 0.024 | 3.542 | 0.037 | 0.130 | 4.01×10^-4^ ** |
|  |  |  |  |  | Total ICV | 0.378 | 0.012 | 32.266 | 0.355 | 0.401 | 1.26×10^-205^ ** |
|  |  |  |  |  | Centre - Reading | -0.085 | 0.035 | -2.449 | -0.154 | -0.017 | .014* |
|  |  |  |  |  | Centre - Newcastle | 0.003 | 0.021 | 0.160 | -0.038 | 0.045 | .873 |
| Amygdala volume (ml) | R^2^ (Conditional) | 0.963 | Participants, SD | 0.31 | (Intercept) | 3.284 | 0.010 | 338.272 | 3.265 | 3.303 | < 1×10^-300^ ** |
|  | R^2^ (Marginal) | 0.428 | ICC, % | 93.5 | **SLE Group** | 0.006 | 0.003 | 1.988 | 8.02×10^-5^ | 0.011 | .047* |
|  |  |  |  |  | **Depression Group** | -0.001 | 0.004 | -0.259 | -0.009 | 0.007 | .795 |
|  |  |  |  |  | Time | -0.012 | 0.001 | -20.060 | -0.014 | -0.011 | 1.14×10^-85^ ** |
|  |  |  |  |  | Age | -0.124 | 0.005 | -25.598 | -0.134 | -0.115 | 4.39×10^-135^ ** |
|  |  |  |  |  | Age^2^ | -0.024 | 0.005 | -5.198 | -0.032 | -0.015 | 2.10×10^-7^ ** |
|  |  |  |  |  | Sex | 0.134 | 0.011 | 11.681 | 0.111 | 0.156 | 4.48×10^-31^ ** |
|  |  |  |  |  | Total ICV | 0.203 | 0.006 | 35.693 | 0.192 | 0.214 | 6.72×10^-246^ ** |
|  |  |  |  |  | Centre - Reading | -0.042 | 0.017 | -2.502 | -0.075 | -0.009 | .012* |
|  |  |  |  |  | Centre - Newcastle | -0.012 | 0.010 | -1.170 | -0.032 | 0.008 | .242 |

**Table S5 *(continued)*.** Model estimates from the exploratory analysis investigating for the effects of depression**.**

| **Brain Structure**  **(Dependent Variable)** |  |  |  |  |  |  |  |  | ***95% CI*** | |  |
| --- | --- | --- | --- | --- | --- | --- | --- | --- | --- | --- | --- |
|  | **Model Fit** |  | **Random Effects** |  | **Fixed Effects** | ***Estimate*** | ***SE*** | ***t*** | ***Lower*** | ***Upper*** | ***P*** |
| Orbitofrontal cortical thickness (mm) | R^2^ (Conditional) | 0.812 | Participants, SD | 0.08 | (Intercept) | 2.484 | 0.003 | 957.487 | 2.479 | 2.489 | < 1×10^-300^ ** |
|  | R^2^ (Marginal) | 0.042 | ICC, % | 80.4 | **SLE Group** | -0.001 | 0.001 | -1.093 | -0.004 | 0.001 | .274 |
|  |  |  |  |  | **Depression Group** | 0.001 | 0.002 | 0.839 | -0.002 | 0.005 | .402 |
|  |  |  |  |  | Time | -0.004 | 2.86×10^-4^ | -13.300 | -0.004 | -0.003 | 1.32×10^-39^ ** |
|  |  |  |  |  | Age | -0.015 | 0.001 | -11.482 | -0.017 | -0.012 | 4.19×10^-30^ ** |
|  |  |  |  |  | Age^2^ | 0.001 | 0.001 | 0.966 | -0.001 | 0.003 | .334 |
|  |  |  |  |  | Sex | 0.024 | 0.003 | 8.055 | 0.018 | 0.030 | 1.01×10^-15^ ** |
|  |  |  |  |  | Total ICV | -0.006 | 0.001 | -4.386 | -0.009 | -0.004 | 1.18×10^-5^ ** |
|  |  |  |  |  | Centre - Reading | -0.006 | 0.004 | -1.286 | -0.014 | 0.003 | .199 |
|  |  |  |  |  | Centre - Newcastle | -0.013 | 0.003 | -4.936 | -0.018 | -0.008 | 8.26×10^-7^ ** |
| Anterior cingulate cortical thickness (mm) | R^2^ (Conditional) | 0.893 | Participants, SD | 0.12 | (Intercept) | 2.527 | 0.004 | 684.386 | 2.520 | 2.535 | < 1×10^-300^ ** |
|  | R^2^ (Marginal) | 0.061 | ICC, % | 88.6 | **SLE Group** | -0.001 | 0.001 | -0.847 | -0.004 | 0.002 | .397 |
|  |  |  |  |  | **Depression Group** | 0.004 | 0.002 | 2.142 | 3.51×10^-4^ | 0.008 | .032* |
|  |  |  |  |  | Time | 4.59×10^-4^ | 3.11×10^-4^ | 1.477 | -1.50×10^-4^ | 0.001 | .140 |
|  |  |  |  |  | Age | 0.009 | 0.002 | 5.042 | 0.006 | 0.013 | 4.80×10^-7^ ** |
|  |  |  |  |  | Age^2^ | 0.007 | 0.002 | 4.135 | 0.004 | 0.010 | 3.62×10^-5^** |
|  |  |  |  |  | Sex | 0.013 | 0.004 | 3.120 | 0.005 | 0.022 | .002 |
|  |  |  |  |  | Total ICV | -0.031 | 0.002 | -14.437 | -0.035 | -0.027 | 3.24×10^-46^ ** |
|  |  |  |  |  | Centre - Reading | -0.014 | 0.006 | -2.238 | -0.027 | -0.002 | .025* |
|  |  |  |  |  | Centre - Newcastle | -0.015 | 0.004 | -3.922 | -0.023 | -0.008 | 8.92×10^-5^ ** |
| Insula cortical thickness (mm) | R^2^ (Conditional) | 0.875 | Participants, SD | 0.11 | (Intercept) | 2.861 | 0.003 | 818.937 | 2.854 | 2.868 | < 1×10^-300^ ** |
|  | R^2^ (Marginal) | 0.011 | ICC, % | 87.4 | **SLE Group** | -0.001 | 0.001 | -0.647 | -0.004 | 0.002 | .518 |
|  |  |  |  |  | **Depression Group** | -0.001 | 0.002 | -0.457 | -0.005 | 0.003 | .648 |
|  |  |  |  |  | Time | -0.003 | 3.09×10^-4^ | -10.088 | -0.004 | -0.003 | 1.13×10^-23^ ** |
|  |  |  |  |  | Age | -0.009 | 0.002 | -5.178 | -0.012 | -0.006 | 2.34×10^-7^ ** |
|  |  |  |  |  | Age^2^ | 0.001 | 0.002 | 0.725 | -0.002 | 0.004 | .469 |
|  |  |  |  |  | Sex | 0.015 | 0.004 | 3.798 | 0.007 | 0.023 | 1.48×10^-4^ ** |
|  |  |  |  |  | Total ICV | -0.002 | 0.002 | -0.787 | -0.006 | 0.002 | .431 |
|  |  |  |  |  | Centre - Reading | 0.007 | 0.006 | 1.216 | -0.004 | 0.019 | .224 |
|  |  |  |  |  | Centre - Newcastle | -0.008 | 0.004 | -2.190 | -0.015 | -0.001 | .029* |

*p < .05; **p < .01. The p-values reported above are uncorrected.

^a^ Variables of interest (highlighted rows) survived false discovery rate (FDR) correction for multiple comparisons.

*Note:* Where the interaction term of SLE group x depression group was not significant, the fixed effects from the model excluding the interaction term are reported above. Only the total GM volume and hippocampus estimates are from the model including the interaction term.

The linear mixed models included participants (*n* = 4,506) modelled as random intercepts. Time is measured in years from the first imaging assessment. All models controlled for sex, age at first imaging assessment, age^2^, total intracranial volume, and scan centre. Age and total ICV were standardised and mean centred to avoid large variation in scales across covariates affecting model convergence.

Depression group (PHQ+ or PHQ-) is based on the cut-off score for the Patient Health Questionnaire (PHQ)-4 depression subscale where scores ≥3 indicate probable depression.

SLE = stressful life event; ICV = intracranial volume; ICC = intraclass correlation coefficient.

Categorical Variables: SLE Group: 0 = SLE-, 1 = SLE+; Depression Group: 0 = PHQ-, 1 = PHQ+; Sex: 0 = Female, 1 = Male; Centre: 0 = Cheadle, 1 = Reading, 2 = Newcastle.

Table S6. Model estimates from the exploratory analysis investigating for the effects of childhood adversity.

| **Brain Structure**  **(Dependent Variable)** |  |  |  |  |  |  |  |  | ***95% CI*** | |  |
| --- | --- | --- | --- | --- | --- | --- | --- | --- | --- | --- | --- |
|  | **Model Fit** |  | **Random Effects** |  | **Fixed Effects** | ***Estimate*** | ***SE*** | ***t*** | ***Lower*** | ***Upper*** | ***P*** |
| Total GM volume (ml) | R^2^ (Conditional) | 0.970 | Participants, SD | 24.08 | (Intercept) | 661.331 | 1.017 | 650.106 | 659.337 | 663.324 | < 1×10^-300^ ** |
|  | R^2^ (Marginal) | 0.781 | ICC, % | 86.4 | **SLE Group** | -0.100 | 0.371 | -0.271 | -0.827 | 0.626 | .786 |
|  |  |  |  |  | **CA Group** | 0.362 | 0.881 | 0.411 | -1.365 | 2.089 | .681 |
|  |  |  |  |  | Time | -1.868 | 0.082 | -22.886 | -2.028 | -1.708 | 6.85×10^-108^ ** |
|  |  |  |  |  | Age | -10.846 | 0.449 | -24.143 | -11.727 | -9.966 | 8.01×10^-119^ ** |
|  |  |  |  |  | Age^2^ | -1.979 | 0.415 | -4.766 | -2.792 | -1.165 | 1.96×10^-6^ ** |
|  |  |  |  |  | Sex | 11.009 | 1.062 | 10.365 | 8.927 | 13.091 | 8.55×10^-25^ ** |
|  |  |  |  |  | Total ICV | 44.159 | 0.524 | 84.248 | 43.132 | 45.186 | < 1×10^-300^ ** |
|  |  |  |  |  | Centre - Reading | -4.672 | 1.561 | -2.992 | -7.732 | -1.612 | .003* |
|  |  |  |  |  | Centre - Newcastle | -1.676 | 0.939 | -1.784 | -3.516 | 0.165 | .074 |
| Hippocampus volume (ml) | R^2^ (Conditional) | 0.967 | Participants, SD | 0.65 | (Intercept) | 8.231 | 0.026 | 310.677 | 8.179 | 8.282 | < 1×10^-300^ ** |
|  | R^2^ (Marginal) | 0.402 | ICC, % | 94.5 | **SLE Group** | 0.004 | 0.006 | 0.703 | -0.008 | 0.017 | .482 |
|  |  |  |  |  | **CA Group** | -2.85×10^-4^ | 0.023 | -0.012 | -0.046 | 0.045 | .990 |
|  |  |  |  |  | Time | -0.051 | 0.001 | -38.018 | -0.054 | -0.048 | 3.51×10^-263^ ** |
|  |  |  |  |  | Age | -0.351 | 0.012 | -29.726 | -0.374 | -0.328 | 2.14×10^-172^ ** |
|  |  |  |  |  | Age^2^ | -0.051 | 0.011 | -4.695 | -0.073 | -0.030 | 2.78×10^-6^ ** |
|  |  |  |  |  | Sex | 0.095 | 0.028 | 3.404 | 0.040 | 0.150 | .001** |
|  |  |  |  |  | Total ICV | 0.377 | 0.014 | 27.354 | 0.350 | 0.404 | 9.99×10^-149^ ** |
|  |  |  |  |  | Centre - Reading | -0.086 | 0.041 | -2.095 | -0.166 | -0.006 | .036* |
|  |  |  |  |  | Centre - Newcastle | -0.002 | 0.025 | -0.093 | -0.051 | 0.046 | .926 |
| Amygdala volume (ml) | R^2^ (Conditional) | 0.963 | Participants, SD | 0.31 | (Intercept) | 3.269 | 0.013 | 253.702 | 3.244 | 3.294 | < 1×10^-300^ ** |
|  | R^2^ (Marginal) | 0.423 | ICC, % | 93.6 | **SLE Group** | 0.005 | 0.003 | 1.425 | -0.002 | 0.011 | .154 |
|  |  |  |  |  | **CA Group** | 0.022 | 0.011 | 1.944 | -1.82×10^-4^ | 0.044 | .052 |
|  |  |  |  |  | Time | -0.013 | 0.001 | -18.410 | -0.014 | -0.012 | 3.57×10^-72^ ** |
|  |  |  |  |  | Age | -0.126 | 0.006 | -21.882 | -0.137 | -0.114 | 2.84×10^-99^ ** |
|  |  |  |  |  | Age^2^ | -0.024 | 0.005 | -4.490 | -0.034 | -0.013 | 7.35×10^-6^ ** |
|  |  |  |  |  | Sex | 0.126 | 0.014 | 9.306 | 0.100 | 0.153 | 2.34×10^-20^ ** |
|  |  |  |  |  | Total ICV | 0.205 | 0.007 | 30.658 | 0.192 | 0.218 | 5.99×10^-182^ ** |
|  |  |  |  |  | Centre - Reading | -0.054 | 0.020 | -2.698 | -0.093 | -0.015 | .007* |
|  |  |  |  |  | Centre - Newcastle | -0.016 | 0.012 | -1.347 | -0.040 | 0.007 | .178 |

**Table S6 *(continued)*.** Model estimates from the exploratory analysis investigating for the effects of childhood adversity.

| **Brain Structure**  **(Dependent Variable)** |  |  |  |  |  |  |  |  | ***95% CI*** | |  |
| --- | --- | --- | --- | --- | --- | --- | --- | --- | --- | --- | --- |
|  | **Model Fit** |  | **Random Effects** |  | **Fixed Effects** | ***Estimate*** | ***SE*** | ***t*** | ***Lower*** | ***Upper*** | ***P*** |
| Orbitofrontal cortical thickness (mm) | R^2^ (Conditional) | 0.817 | Participants, SD | 0.08 | (Intercept) | 2.483 | 0.003 | 732.012 | 2.477 | 2.490 | < 1×10^-300^ ** |
|  | R^2^ (Marginal) | 0.040 | ICC, % | 80.9 | **SLE Group** | -0.002 | 0.001 | -1.122 | -0.004 | 0.001 | .262 |
|  |  |  |  |  | **CA Group** | 0.004 | 0.003 | 1.497 | -0.001 | 0.010 | .134 |
|  |  |  |  |  | Time | -0.004 | 3.25×10^-4^ | -12.656 | -0.005 | -0.003 | 6.57×10^-36^ ** |
|  |  |  |  |  | Age | -0.014 | 0.001 | -9.402 | -0.017 | -0.011 | 9.63×10^-21^ ** |
|  |  |  |  |  | Age^2^ | 0.002 | 0.001 | 1.486 | -0.001 | 0.005 | .137 |
|  |  |  |  |  | Sex | 0.022 | 0.004 | 6.208 | 0.015 | 0.029 | 6.04×10^-10^ ** |
|  |  |  |  |  | Total ICV | -0.005 | 0.002 | -3.161 | -0.009 | -0.002 | .002** |
|  |  |  |  |  | Centre - Reading | -0.005 | 0.005 | -0.876 | -0.015 | 0.006 | .381 |
|  |  |  |  |  | Centre - Newcastle | -0.015 | 0.003 | -4.925 | -0.021 | -0.009 | 8.87×10^-7^ ** |
| Anterior cingulate cortical thickness (mm) | R^2^ (Conditional) | 0.892 | Participants, SD | 0.12 | (Intercept) | 2.523 | 0.005 | 518.352 | 2.513 | 2.532 | < 1×10^-300^ ** |
|  | R^2^ (Marginal) | 0.067 | ICC, % | 88.5 | **SLE Group** | -0.001 | 0.002 | -0.495 | -0.004 | 0.002 | .621 |
|  |  |  |  |  | **CA Group** | 0.001 | 0.004 | 0.149 | -0.008 | 0.009 | .882 |
|  |  |  |  |  | Time | 0.001 | 3.59×10^-4^ | 1.556 | -1.45×10^-4^ | 0.001 | .120 |
|  |  |  |  |  | Age | 0.010 | 0.002 | 4.479 | 0.005 | 0.014 | 7.73×10^-6^ ** |
|  |  |  |  |  | Age^2^ | 0.010 | 0.002 | 5.117 | 0.006 | 0.014 | 3.29×10^-7^ ** |
|  |  |  |  |  | Sex | 0.017 | 0.005 | 3.399 | 0.007 | 0.027 | .001** |
|  |  |  |  |  | Total ICV | -0.032 | 0.003 | -12.817 | -0.037 | -0.027 | 9.63×10^-37^ ** |
|  |  |  |  |  | Centre - Reading | -0.010 | 0.007 | -1.350 | -0.025 | 0.005 | .177 |
|  |  |  |  |  | Centre - Newcastle | -0.017 | 0.005 | -3.679 | -0.025 | -0.008 | 2.38×10^-4^ ** |
| Insula cortical thickness (mm) | R^2^ (Conditional) | 0.875 | Participants, SD | 0.11 | (Intercept) | 2.860 | 0.005 | 616.169 | 2.851 | 2.869 | < 1×10^-300^ ** |
|  | R^2^ (Marginal) | 0.012 | ICC, % | 87.4 | **SLE Group** | -0.002 | 0.002 | -1.205 | -0.005 | 0.001 | .228 |
|  |  |  |  |  | **CA Group** | 0.002 | 0.004 | 0.566 | -0.006 | 0.010 | .572 |
|  |  |  |  |  | Time | -0.003 | 3.59×10^-4^ | -8.892 | -0.004 | -0.002 | 9.55×10^-19^ ** |
|  |  |  |  |  | Age | -0.009 | 0.002 | -4.402 | -0.013 | -0.005 | 1.11×10^-5^ ** |
|  |  |  |  |  | Age^2^ | 0.002 | 0.002 | 1.142 | -0.002 | 0.006 | .254 |
|  |  |  |  |  | Sex | 0.016 | 0.005 | 3.269 | 0.006 | 0.025 | .001** |
|  |  |  |  |  | Total ICV | -0.002 | 0.002 | -0.649 | -0.006 | 0.003 | .516 |
|  |  |  |  |  | Centre - Reading | 0.008 | 0.007 | 1.090 | -0.006 | 0.022 | .276 |
|  |  |  |  |  | Centre - Newcastle | -0.011 | 0.004 | -2.551 | -0.019 | -0.003 | .011* |

*p < .05; **p < .01. The p-values reported above are uncorrected.

^a^ Variables of interest (highlighted rows) survived false discovery rate (FDR) correction for multiple comparisons.

*Note:* As the interaction term CA group x SLE group was not significant across all regions, the fixed effects from the model excluding the interaction term are reported above.

The linear mixed models included participants (*n* = 3,324) modelled as random intercepts. Time is measured in years from the first imaging assessment. All models controlled for sex, age at first imaging assessment, age^2^, total intracranial volume, and scan centre. Age and total ICV were standardised and mean centred to avoid large variation in scales across covariates affecting model convergence.

Childhood adversity group is based on scores from the Childhood Trauma Screener (CTS)-5, where participants were grouped into no childhood adversity (CA-), with scores of zero, or any childhood adversity (CA+), with scores of more than zero.

SLE = stressful life event; CA = childhood adversity; ICV = intracranial volume; ICC = intraclass correlation coefficient.

Categorical Variables: SLE Group: 0 = SLE-, 1 = SLE+; CA Group: 0 = CA-, 1 = CA+; Sex: 0 = Female, 1 = Male; Centre: 0 = Cheadle, 1 = Reading, 2 = Newcastle.

Table S7. SLE group estimates across all brain structures.

|  |  | **SLE Group Estimate**^1^ | **95% CI** | |  |  |
| --- | --- | --- | --- | --- | --- | --- |
| **Brain Regions** | **Hemisphere** |  | **Lower** | **Upper** | ***P*** | ***P* (corrected)** |
| ***Cortical Thickness*** |  |  |  |  |  |  |
| Banks of the Superior Temporal Sulcus | L | -0.004 | -0.008 | -0.001 | **0.013** | 0.504 |
|  | R | -0.003 | -0.007 | 0.000 | 0.060 | 0.762 |
| Caudal Anterior Cingulate | L | -0.003 | -0.007 | 0.001 | 0.124 | 0.762 |
|  | R | -0.003 | -0.006 | 0.001 | 0.104 | 0.762 |
| Caudal Middle Frontal | L | 0.000 | -0.005 | 0.006 | 0.938 | 0.988 |
|  | R | 0.001 | -0.004 | 0.006 | 0.765 | 0.988 |
| Cuneus | L | 0.000 | -0.003 | 0.002 | 0.870 | 0.988 |
|  | R | 0.000 | -0.003 | 0.003 | 0.994 | 0.999 |
| Entorhinal | L | -0.003 | -0.009 | 0.004 | 0.410 | 0.988 |
|  | R | -0.005 | -0.011 | 0.001 | 0.100 | 0.762 |
| Frontal Pole | L | 0.006 | -0.003 | 0.015 | 0.207 | 0.988 |
|  | R | 0.001 | -0.008 | 0.010 | 0.821 | 0.988 |
| Fusiform | L | -0.001 | -0.004 | 0.002 | 0.357 | 0.988 |
|  | R | -0.004 | -0.007 | -0.001 | **0.004** | 0.329 |
| Inferior Parietal | L | -0.001 | -0.005 | 0.004 | 0.823 | 0.988 |
|  | R | -0.001 | -0.007 | 0.004 | 0.659 | 0.988 |
| Inferior Temporal | L | -0.003 | -0.007 | 0.000 | 0.070 | 0.762 |
|  | R | -0.002 | -0.006 | 0.002 | 0.279 | 0.988 |
| Insula | L | -0.001 | -0.004 | 0.003 | 0.713 | 0.988 |
|  | R | -0.003 | -0.006 | 0.000 | 0.090 | 0.762 |
| Isthmus Cingulate | L | 0.001 | -0.002 | 0.004 | 0.393 | 0.988 |
|  | R | 0.000 | -0.003 | 0.003 | 0.999 | 0.999 |
| Lateral Occipital | L | -0.002 | -0.007 | 0.002 | 0.295 | 0.988 |
|  | R | -0.002 | -0.007 | 0.004 | 0.556 | 0.988 |
| Lateral Orbitofrontal | L | -0.003 | -0.006 | 0.000 | 0.088 | 0.762 |
|  | R | -0.002 | -0.005 | 0.002 | 0.377 | 0.988 |
| Lingual | L | 0.002 | -0.001 | 0.005 | 0.202 | 0.988 |
|  | R | 0.002 | -0.001 | 0.005 | 0.140 | 0.768 |
| Medial Orbitofrontal | L | -0.001 | -0.003 | 0.002 | 0.730 | 0.988 |
|  | R | -0.001 | -0.005 | 0.002 | 0.359 | 0.988 |
| Middle Temporal | L | -0.002 | -0.007 | 0.003 | 0.343 | 0.988 |
|  | R | -0.004 | -0.009 | 0.001 | 0.104 | 0.762 |
| Paracentral | L | 0.002 | -0.002 | 0.006 | 0.339 | 0.988 |
|  | R | 0.001 | -0.003 | 0.005 | 0.597 | 0.988 |
| Parahippocampal | L | 0.001 | -0.003 | 0.005 | 0.664 | 0.988 |
|  | R | -0.003 | -0.007 | 0.001 | 0.118 | 0.762 |
| Pars Opercularis | L | 0.000 | -0.005 | 0.004 | 0.823 | 0.988 |
|  | R | -0.006 | -0.010 | -0.002 | **0.004** | 0.329 |
| Pars Orbitalis | L | 0.001 | -0.005 | 0.007 | 0.786 | 0.988 |
|  | R | -0.005 | -0.010 | 0.001 | 0.109 | 0.762 |
| Pars Triangularis | L | 0.001 | -0.004 | 0.006 | 0.695 | 0.988 |
|  | R | -0.004 | -0.008 | 0.001 | 0.129 | 0.762 |
| Pericalcarine | L | 0.002 | -0.001 | 0.006 | 0.227 | 0.988 |
|  | R | 0.004 | 0.000 | 0.008 | **0.037** | 0.641 |
| Postcentral | L | 0.000 | -0.003 | 0.004 | 0.844 | 0.988 |
|  | R | 0.000 | -0.004 | 0.004 | 0.996 | 0.999 |
| Posterior Cingulate | L | 0.002 | -0.001 | 0.004 | 0.281 | 0.988 |
|  | R | 0.000 | -0.003 | 0.003 | 0.841 | 0.988 |
| Precentral | L | -0.002 | -0.008 | 0.005 | 0.609 | 0.988 |
|  | R | -0.002 | -0.008 | 0.004 | 0.479 | 0.988 |
| Precuneus | L | -0.001 | -0.004 | 0.002 | 0.614 | 0.988 |
|  | R | -0.004 | -0.007 | -0.001 | **0.013** | 0.504 |
| Rostral Anterior Cingulate | L | 0.002 | -0.002 | 0.006 | 0.357 | 0.988 |
|  | R | -0.002 | -0.006 | 0.003 | 0.405 | 0.988 |

**Table S7 *(continued)*.** SLE group estimates across all brain structures.

|  |  | **SLE Group Estimate**^1^ | **95% CI** | |  |  |
| --- | --- | --- | --- | --- | --- | --- |
| **Brain Regions** | **Hemisphere** |  | **Lower** | **Upper** | **P** | **P (corrected)** |
| ***Cortical Thickness*** |  |  |  |  |  |  |
| Rostral Middle Frontal | L | 0.001 | -0.003 | 0.006 | 0.577 | 0.988 |
|  | R | 0.000 | -0.005 | 0.004 | 0.849 | 0.988 |
| Superior Frontal | L | 0.001 | -0.003 | 0.004 | 0.731 | 0.988 |
|  | R | 0.001 | -0.003 | 0.004 | 0.705 | 0.988 |
| Superior Parietal | L | 0.000 | -0.004 | 0.003 | 0.878 | 0.988 |
|  | R | 0.001 | -0.003 | 0.004 | 0.760 | 0.988 |
| Superior Temporal | L | -0.002 | -0.006 | 0.001 | 0.228 | 0.988 |
|  | R | -0.003 | -0.006 | 0.001 | 0.130 | 0.762 |
| Supramarginal | L | 0.000 | -0.005 | 0.004 | 0.962 | 0.988 |
|  | R | -0.001 | -0.006 | 0.004 | 0.658 | 0.988 |
| Temporal Pole | L | -0.004 | -0.011 | 0.003 | 0.254 | 0.988 |
|  | R | -0.004 | -0.011 | 0.004 | 0.371 | 0.988 |
| Transverse Temporal | L | -0.001 | -0.005 | 0.004 | 0.790 | 0.988 |
|  | R | -0.001 | -0.006 | 0.004 | 0.600 | 0.988 |
| ***Surface Area*** |  |  |  |  |  |  |
| Banks of the Superior Temporal Sulcus | L | -0.328 | -2.009 | 1.352 | 0.702 | 0.988 |
|  | R | -0.319 | -1.432 | 0.795 | 0.575 | 0.988 |
| Caudal Anterior Cingulate | L | 0.099 | -0.781 | 0.978 | 0.826 | 0.988 |
|  | R | 0.373 | -0.632 | 1.378 | 0.467 | 0.988 |
| Caudal Middle Frontal | L | -4.024 | -12.512 | 4.464 | 0.353 | 0.988 |
|  | R | -3.248 | -10.832 | 4.336 | 0.401 | 0.988 |
| Cuneus | L | -1.308 | -3.501 | 0.885 | 0.242 | 0.988 |
|  | R | -1.880 | -4.335 | 0.576 | 0.134 | 0.762 |
| Entorhinal | L | 0.462 | -0.833 | 1.757 | 0.484 | 0.988 |
|  | R | 0.335 | -0.778 | 1.448 | 0.556 | 0.988 |
| Frontal Pole | L | -0.091 | -2.701 | 2.518 | 0.945 | 0.988 |
|  | R | 0.456 | -2.934 | 3.847 | 0.792 | 0.988 |
| Fusiform | L | -0.135 | -2.930 | 2.660 | 0.924 | 0.988 |
|  | R | -0.591 | -3.415 | 2.233 | 0.682 | 0.988 |
| Inferior Parietal | L | 1.165 | -16.616 | 18.945 | 0.898 | 0.988 |
|  | R | -8.350 | -32.525 | 15.826 | 0.498 | 0.988 |
| Inferior Temporal | L | -0.322 | -9.336 | 8.691 | 0.944 | 0.988 |
|  | R | -0.700 | -11.634 | 10.233 | 0.900 | 0.988 |
| Insula | L | -1.175 | -4.075 | 1.724 | 0.427 | 0.988 |
|  | R | -1.254 | -3.894 | 1.386 | 0.352 | 0.988 |
| Isthmus Cingulate | L | -0.742 | -2.323 | 0.840 | 0.358 | 0.988 |
|  | R | -0.461 | -1.956 | 1.033 | 0.545 | 0.988 |
| Lateral Occipital | L | 1.818 | -16.142 | 19.778 | 0.843 | 0.988 |
|  | R | -4.880 | -24.790 | 15.030 | 0.631 | 0.988 |
| Lateral Orbitofrontal | L | -1.746 | -6.507 | 3.015 | 0.472 | 0.988 |
|  | R | -5.417 | -10.670 | -0.165 | **0.043** | 0.667 |
| Lingual | L | -0.261 | -4.724 | 4.201 | 0.909 | 0.988 |
|  | R | -2.812 | -7.587 | 1.963 | 0.248 | 0.988 |
| Medial Orbitofrontal | L | -3.317 | -7.478 | 0.844 | 0.118 | 0.762 |
|  | R | -4.441 | -8.528 | -0.354 | **0.033** | 0.640 |
| Middle Temporal | L | 1.177 | -12.811 | 15.165 | 0.869 | 0.988 |
|  | R | -0.946 | -15.981 | 14.089 | 0.902 | 0.988 |
| Paracentral | L | -1.281 | -3.619 | 1.056 | 0.283 | 0.988 |
|  | R | -0.661 | -3.346 | 2.024 | 0.630 | 0.988 |
| Parahippocampal | L | -0.940 | -1.748 | -0.131 | **0.023** | 0.640 |
|  | R | -0.230 | -0.877 | 0.418 | 0.487 | 0.988 |
| Pars Opercularis | L | -2.505 | -7.225 | 2.214 | 0.298 | 0.988 |
|  | R | 1.189 | -2.033 | 4.411 | 0.470 | 0.988 |

**Table S7 *(continued)*.** SLE group estimates across all brain structures.

|  |  | **SLE Group Estimate**^1^ | **95% CI** | |  |  |
| --- | --- | --- | --- | --- | --- | --- |
| **Brain Regions** | **Hemisphere** |  | **Lower** | **Upper** | **P** | **P (corrected)** |
| ***Surface Area*** |  |  |  |  |  |  |
| Pars Orbitalis | L | -0.170 | -3.528 | 3.188 | 0.921 | 0.988 |
|  | R | 1.239 | -2.682 | 5.160 | 0.536 | 0.988 |
| Pars Triangularis | L | -0.733 | -6.640 | 5.174 | 0.808 | 0.988 |
|  | R | 1.938 | -4.649 | 8.525 | 0.564 | 0.988 |
| Pericalcarine | L | 0.448 | -1.040 | 1.936 | 0.555 | 0.988 |
|  | R | 0.279 | -1.315 | 1.872 | 0.732 | 0.988 |
| Postcentral | L | 2.064 | -9.082 | 13.210 | 0.717 | 0.988 |
|  | R | 1.084 | -9.416 | 11.584 | 0.840 | 0.988 |
| Posterior Cingulate | L | -0.867 | -2.083 | 0.349 | 0.162 | 0.862 |
|  | R | -0.123 | -1.431 | 1.184 | 0.854 | 0.988 |
| Precentral | L | -1.427 | -17.037 | 14.183 | 0.858 | 0.988 |
|  | R | 2.393 | -12.607 | 17.393 | 0.755 | 0.988 |
| Precuneus | L | -0.093 | -3.653 | 3.467 | 0.959 | 0.988 |
|  | R | 2.783 | -0.778 | 6.344 | 0.126 | 0.762 |
| Rostral Anterior Cingulate | L | -0.250 | -1.729 | 1.230 | 0.741 | 0.988 |
|  | R | -0.022 | -1.208 | 1.165 | 0.971 | 0.990 |
| Rostral Middle Frontal | L | -0.977 | -27.660 | 25.706 | 0.943 | 0.988 |
|  | R | -4.658 | -30.805 | 21.490 | 0.727 | 0.988 |
| Superior Frontal | L | 0.563 | -19.332 | 20.458 | 0.956 | 0.988 |
|  | R | -5.028 | -23.157 | 13.100 | 0.587 | 0.988 |
| Superior Parietal | L | 4.500 | -11.853 | 20.853 | 0.590 | 0.988 |
|  | R | 1.586 | -15.311 | 18.482 | 0.854 | 0.988 |
| Superior Temporal | L | -2.460 | -12.719 | 7.800 | 0.638 | 0.988 |
|  | R | -2.690 | -11.443 | 6.063 | 0.547 | 0.988 |
| Supramarginal | L | 1.318 | -14.218 | 16.853 | 0.868 | 0.988 |
|  | R | 1.197 | -12.213 | 14.608 | 0.861 | 0.988 |
| Temporal Pole | L | 0.364 | -1.551 | 2.279 | 0.710 | 0.988 |
|  | R | -1.660 | -3.504 | 0.184 | 0.078 | 0.762 |
| Transverse Temporal | L | -0.121 | -1.059 | 0.817 | 0.800 | 0.988 |
|  | R | -0.270 | -1.068 | 0.528 | 0.508 | 0.988 |
| ***Subcortical Volume*** |  |  |  |  |  |  |
| Amygdala | L | 0.002 | -0.002 | 0.005 | 0.371 | 0.988 |
|  | R | 0.003 | -0.001 | 0.006 | 0.115 | 0.762 |
| Caudate | L | 0.001 | -0.006 | 0.007 | 0.849 | 0.988 |
|  | R | 0.001 | -0.007 | 0.008 | 0.884 | 0.988 |
| Cerebellum | L | -0.005 | -0.095 | 0.084 | 0.904 | 0.988 |
|  | R | 0.003 | -0.086 | 0.092 | 0.950 | 0.988 |
| Hippocampus | L | 0.000 | -0.006 | 0.006 | 0.943 | 0.988 |
|  | R | 0.000 | -0.005 | 0.006 | 0.895 | 0.988 |
| Lateral Ventricles | L | -0.007 | -0.060 | 0.046 | 0.808 | 0.988 |
|  | R | 0.017 | -0.031 | 0.066 | 0.482 | 0.988 |
| Nucleus Accumbens | L | 0.001 | -0.004 | 0.005 | 0.805 | 0.988 |
|  | R | 0.001 | -0.002 | 0.004 | 0.536 | 0.988 |
| Pallidum | L | 0.005 | 0.000 | 0.009 | **0.030** | 0.640 |
|  | R | 0.005 | 0.001 | 0.009 | **0.026** | 0.640 |
| Putamen | L | 0.008 | -0.007 | 0.023 | 0.275 | 0.988 |
|  | R | 0.002 | -0.010 | 0.015 | 0.704 | 0.988 |
| Thalamus | L | -0.003 | -0.015 | 0.010 | 0.678 | 0.988 |
|  | R | -0.002 | -0.016 | 0.012 | 0.776 | 0.988 |

Uncorrected *p* < .05 are in **bold**. Findings were corrected for multiple comparisons using the 5% false discovery rate method.

SLE = stressful life event; Categorical Variables: SLE Group: 0 = SLE-, 1 = SLE+.

^1^ Each brain structure was modelled in a separate linear mixed model as the dependent variable, with participants (*n* = 4,543) modelled as random intercepts, and SLE group as the main fixed effect. All models controlled for time (years from first imaging assessment), sex, age at first imaging assessment, age^2^, total intracranial volume, and scan centre. Age and total ICV were standardised and mean centred to avoid large variation in scales across covariates affecting model convergence.

Supplementary References

1. Kroenke K, Spitzer RL, Williams JBW, Löwe B. An Ultra-Brief Screening Scale for Anxiety and Depression: The PHQ–4. Psychosomatics. 2009;50(6):613-21. 10.1016/s0033-3182(09)70864-3

2. Lowe B, Wahl I, Rose M, Spitzer C, Glaesmer H, Wingenfeld K, et al. A 4-item measure of depression and anxiety: validation and standardization of the Patient Health Questionnaire-4 (PHQ-4) in the general population. J Affect Disord. 2010;122(1-2):86-95. 10.1016/j.jad.2009.06.019

3. Townsend P, Phillimore P, Beattie A. Health and Deprivation: Inequality and the North. London: Routledge; 1988.

4. Littmann A, Guehring J, Buechel C, Stiehl HS. Acquisition-related morphological variability in structural MRI. Acad Radiol. 2006;13(9):1055-61. 10.1016/j.acra.2006.05.001

5. Alfaro-Almagro F, McCarthy P, Afyouni S, Andersson JLR, Bastiani M, Miller KL, et al. Confound modelling in UK Biobank brain imaging. Neuroimage. 2021;224:117002. 10.1016/j.neuroimage.2020.117002
